# The Role of Tumor Microenvironment Cells in Colorectal Cancer (CRC) Cachexia

**DOI:** 10.3390/ijms22041565

**Published:** 2021-02-04

**Authors:** Aldona Kasprzak

**Affiliations:** Department of Histology and Embryology, University of Medical Sciences, Święcicki Street 6, 60-781 Poznań, Poland; akasprza@ump.edu.pl; Tel.: +48-61-8546441; Fax: +48-61-8546440

**Keywords:** tumor microenvironment, colorectal cancer, cancer cachexia, stromal and cancer cells, cachexia-inducing factors, pro-inflammatory cytokines

## Abstract

Cancer cachexia (CC) is a multifactorial syndrome in patients with advanced cancer characterized by weight loss via skeletal-muscle and adipose-tissue atrophy, catabolic activity, and systemic inflammation. CC is correlated with functional impairment, reduced therapeutic responsiveness, and poor prognosis, and is a major cause of death in cancer patients. In colorectal cancer (CRC), cachexia affects around 50–61% of patients, but remains overlooked, understudied, and uncured. The mechanisms driving CC are not fully understood but are related, at least in part, to the local and systemic immune response to the tumor. Accumulating evidence demonstrates a significant role of tumor microenvironment (TME) cells (e.g., macrophages, neutrophils, and fibroblasts) in both cancer progression and tumor-induced cachexia, through the production of multiple procachectic factors. The most important role in CRC-associated cachexia is played by pro-inflammatory cytokines, including the tumor necrosis factor α (TNFα), originally known as cachectin, Interleukin (IL)-1, IL-6, and certain chemokines (e.g., IL-8). Heterogeneous CRC cells themselves also produce numerous cytokines (including chemokines), as well as novel factors called “cachexokines”. The tumor microenvironment (TME) contributes to systemic inflammation and increased oxidative stress and fibrosis. This review summarizes the current knowledge on the role of TME cellular components in CRC-associated cachexia, as well as discusses the potential role of selected mediators secreted by colorectal cancer cells in cooperation with tumor-associated immune and non-immune cells of tumor microenvironment in inducing or potentiating cancer cachexia. This knowledge serves to aid the understanding of the mechanisms of this process, as well as prevent its consequences.

## 1. Introduction

Cancer cachexia (CC) is a multifactorial clinical complication in numerous human malignancies. Its incidence in Europe is highest in patients with lung (83/36% inpatients/outpatients, respectively) and gastrointestinal cancers (62/42%) [1]. There are currently no therapeutic agents used for treatment of this paraneoplastic syndrome, caused by impaired/reduced nutritional intake, or metabolic disorders with increased catabolism and chronic inflammation [2]. In the mechanisms of CC pathogenesis, in addition to inflammation, the role of insulin resistance, damage to mitochondrial function, oxidative stress, as well as the activation of lipolysis and proteolysis through the ubiquitin-proteasome system and macro-autophagy are emphasized (reviewed in: [3]). The main target in CC is skeletal [4,5,6,7] and cardiac muscle [8,9], as well as the adipose tissue (AT) [10].

In case of colorectal cancer (CRC), which is the third most commonly diagnosed malignancy and the second leading cause of cancer death [11], the prevalence of cachexia varies between 50–61%, and is responsible for the death of at least 20% of cancer patients [12,13,14]. Both reduced muscle mass (myopenia), and infiltration by intermuscular and intramuscular fat (myosteatosis) were identified as independent prognostic factors for shorter cancer-specific survival (CSS), overall survival (OS), and disease-free survival (DFS) following curative CRC resection [15,16].

In cancer cachexia, a participation of the tumor microenvironment (TME) [17,18,19,20] and macroenvironment is often underlined [21]. The TME, as a complex cellular network, is constructed of both cancer cells, various stromal cell populations, e.g., infiltrating immune cells (lymphocytes and macrophages) [17,19], neutrophils [20,22,23], fibroblasts [17,19,23], and adipocytes [18], as well as extracellular-matrix (ECM) components, soluble factors, and signaling molecules produced by these cells [17,18,19,20,22,23].

TME-associated tumor–host interactions have been demonstrated to contribute to plasma cytokine level and proteome alterations, possibly assisting the development of CC, as well as tumor progression [24]. The source of these cytokines and their final effect on the organism are still under investigation and are likely multifactorial. The cachexia-inducing factors (CIFs) include tumor necrosis factor α (TNF-α), Interleukin 1 and 6 (IL-1, IL-6), Interferon γ (Interferon γ) [25,26], selected hormones (e.g., angiotensin II, glucocorticoids, ghrelin, and leptin) [25,27], and other factors (e.g., myostatin) [27], produced by both tumors, e.g., proteolysis-inducing factor (PIF) and host cells (e.g., TNF-α, and IL-6) [25,26]. Moreover, profiling of tumor gene expression of CIFs in human cancers characterized with varying cachexia prevalence was also performed [28].

The TME participates in immune cell activation and recruitment, angiogenesis, and ECM remodeling, all of which result in tumor progression (including CRC) [6,19]. Furthermore, the TME is also a site of local inflammation and contributes to systemic inflammation, increased oxidative stress, and AT fibrosis in cachectic gastrointestinal cancer patients [29,30]. The molecular mechanisms of the cancer-cachexia development involving TME cells are still poorly understood in colorectal cancer. The recently performed single-cell transcriptional analysis of TME composition and characteristics in CRC liver metastases identified a total of 12 clusters corresponding to six cell types, including cancer cells, T cells, myeloid cells, endothelial cells (ECs), fibroblasts, and B cells [31]. Moreover, it is known that the molecular classification of CRC tumors affects TME, e.g., CRCs of high microsatellite instability (MSI-H). Hypermutated or consensus molecular subtype 1 (CMS 1) tumors are often infiltrated by lymphocytes with antitumor activity (reviewed in: [32]).

This review summarizes the current knowledge on the role of TME cellular components in CRC-associated cachexia, as well as discusses the potential role of selected mediators secreted by colorectal cancer cells in cooperation with tumor-associated immune and non-immune cells of the TME in inducing or potentiating cancer cachexia. This knowledge serves to aid the understanding of the mechanisms of this process, as well as prevent its consequences.

## 2. Criteria of Cancer Cachexia

A great deal of effort has been devoted to defining and describing CC criteria [14]. Based on SCRINIO Working Group classification, different stages can be indicated, with different severity of CC, from asymptomatic precachexia (class 1) to symptomatic cachexia (class 4) [33]. According to a group of experts participating in a formal international consensus process (2011), CC can be described as a multifactorial syndrome of continuous skeletal-muscle loss, with or without the loss of adipose-tissue mass, functional disorders and a varying extent of appetite impairment that cannot be fully reversed by usual means of nutritional support. It has been confirmed that cachexia develops gradually, through different stages, from precachexia through cachexia to refractory cachexia, with the diagnostic criteria and domains of clinical management of these stages determined [34]. In the recent years, updated criteria were indicated for advanced cancer patients, based on a cachexia staging score (CSS) that consists of five components (weight loss, muscle function, appetite, performance status, and abnormal biochemistry), in order to clarify the above-mentioned three-level staging system (precachexia, cachexia, and refractory cachexia) [35]. Other authors also note that cachexia was connected to higher susceptibility to toxicities associated with treatment, decrease in life quality, and increase in cancer-related mortality, as well as a decrease in cancer-chemotherapy efficacy [2,14]. Other clinical characteristics of cachexia include disrupted metabolism, inflammation, and anorexia, resulting in a persistent fatigue [12], as well as systemic reprogramming of the host metabolism [21]. Furthermore, hypermetabolic patients exhibit more severe and more frequent inflammatory response (higher C-reactive protein (CRP) concentrations), as compared with normometabolic/hypometabolic patients. CRP concentrations, energy intakes, and hypermetabolism, as independent variables, were associated with risk of weight loss >5% [36].

In clinical practice, further studies are needed to unify the criteria for the nutritional status of cachexia. The cancer cachexia study group (CCSG) scoring system (0–3) was indicated as the best OS prognostic evaluator in CRC patients from Norway and Canada. In this system, CC is defined by either a weight loss ≥ 5% during the last 6 months, a weight loss 2–5% in combination with a BMI < 20, or a weight loss of 2–5% with the presence of sarcopenia. Cachexia (in 22–55% patients) and malnutrition (in 34% patients) were indicated as independent significant predictors of survival, after adjusting for nationality, age, and gender [37]. In turn, malnutrition status was observed in 30–60% of Portuguese patients with CRC [38]. Moreover, the study of basal nutritional status in patients with CRC qualified for chemotherapy in Poland, based on the SCRINIO Working Group classification, showed that the majority of them (75%) exhibit precachexia status [39].

## 3. Cancer Cachexia and Systemic Inflammatory Response (SIR)

CC is a secondary disease developing in cancer patients, causing progressive disfunction due to systemic inflammatory response (SIR) [14] or is simply called an “inflammatory condition” [27]. Host–tumor interaction associated SIR was indicated as the seventh hallmark of cancer [40]. SIR is characterized by an increase in concentration of inflammatory factors, such as CRP, TNF-α, IL-1, IL-6, INF-γ, and PIF (previously known as “cancer cachectic factor” or cachexia-associated protein) [27,29,41,42]. Hence, while TNF-α is considered a classical wasting-associated “cachectin” [43], the list of procachectic mediators is continuously expanded. This section of the paper concisely characterizes the factors that have a proven role in cachexia induction.

### 3.1. Tumor Necrosis Factor α (TNF-α) and Proteolysis-Inducing Factor (PIF)

The early review papers already indicate the ability of macrophage produced TNF-α to induce cachexia [43].

As per the results of the pioneering studies on the causes of muscle wasting in CC, TNF-α and IFN-γ strongly suppress the expression of myosin via RNA-dependent mechanism in myotubes and mouse muscle tissue. In the mouse model of the colon-C26 tumor, it was confirmed that the reduction of this protein was associated with the ubiquitin-dependent proteasome (UPS) pathway [44]. TNF-α-mediated inhibition of protein synthesis occurs through the reduction in the active eukaryotic initiation-factor 4E (eIF4E) complex. TNF-α plays a direct role in cachexia, causing catabolic effects not only in muscles but also in AT through the inhibition of lipoprotein lipase, resulting in a loss of AT. Furthermore, this cytokine increases gluconeogenesis and glycogen synthesis [5,41]. In contrast to TNF-α, sulphated glycoprotein PIF only exerts its effects on the muscle tissue, with inhibition of protein synthesis occurring due to increased phosphorylation of the eIF2 on the alpha-subunit. Serum concentrations of TNF-α do not correlate with weight loss, whereas PIF is detectable in the urine, making it a useful marker of weight loss in cachectic patients [41]. Binding of PIF to its receptor in skeletal muscles causes an increase in Ca^+2^_i_, initiating a signaling cascade that leads to a decrease in protein synthesis and increase in protein degradation [45]. Thus, CC is accompanied by an increase in pro-inflammatory factors, including TNF-α and PIF. The main effects of both of these proteins in the context of CC are muscle atrophy caused by decreased protein synthesis and an increase in protein degradation [41]. Additional effects of TNF-α include decreased food intake and increased energy expenditure (reviewed in: [26,27]). Furthermore, other actions of these factors are presented in the chapter considering the involvement of TME cells in CC.

### 3.2. Interleukin 1 α and β (IL-1α, IL-1β)

IL-1α is also considered a key mediator in CC, with the mechanisms of its action discussed in recent reviews in the context of possible therapy [46]. While the main source of IL-1β are the innate immune cells, IL-1α synthesis is localized in various cell types under physiological and pathological conditions (e.g., innate and adaptive immune cells, epithelial cells, ECs, adipocytes, chondrocytes, and fibroblasts) [47]. Furthermore, it needs to be noted that the pleiotropic IL-1 consists of two antagonistic IL-1α and IL-1β cytokines that bind to the same receptor (IL-1R1) and induce the same biological functions. However, both of these proteins show biologically different roles in carcinogenesis. IL-1α’s action is mainly immunostimulatory, while IL-1β exhibits a pro-inflammatory role, especially in the early phases of tumor development [47,48]. In TME, IL-1 is produced by the tumor, as well as stromal and infiltrating cells supporting cancer progression [48]. Considering the subcellular localization, IL-1α can be mostly found inside the cells, both in cytosol and the nucleus, as well as on the cell membrane, rarely being expressed outside the cell. The IL-1α (proIL-1α) precursor form is active and mainly located in cytosol or on cell membranes [49]. In turn, IL-1β is only active as a mature secreted molecule, mainly produced by activated myeloid cells. In the early stages of carcinogenesis, both forms of IL-1 serve a different role. The membrane-bound IL-1α exhibits a mainly immunostimulatory activity, while IL-1β in the TME exhibits a pro-inflammatory role in tumorigenesis and tumor-invasion-promoting activity, as well as immunosuppressive activity [48]. The activity of IL-1R1, present in numerous TME cells, suggests the participation of IL-1 signaling in various stages of tumor development. The mechanisms of IL-1/IL-1R action in TME cells were thoroughly presented in a recent excellent review [47]. The role of IL-1 in tumor progression and its potential in antitumor immunotherapies have been also reviewed by others [49].

While there are some studies on the processes involving the action of both IL-1 agonistic molecules in tissue wasting, the direct mechanisms in human CC tissue wasting are not recognized [7,42,50]. Using the mature C2C12 myotubes model, it was proven that both IL-1 isoforms (α and β) act through an oxidant and serine-threonine protein kinase Akt/Forkhead-O (FOXO)-independent mechanism to activate p38 MAPK, stimulate nuclear factor kappa B (NF-κB) signaling, increase the expression of muscle-specific ubiquitin ligases, atrogin 1/muscle atrophy F-box (MAFbx) and muscle RING-finger 1 (MuRF1), and reduce myofibrillar protein in differentiated myotubes. The authors suggest that direct exposure of muscle cells to IL-1 promotes the expression of E3 ligases and stimulates the catabolism of myotubes, without the participation of reactive oxygen species (ROS) or Akt/FOXO signaling [50].

In a rodent model of cancer cachexia, a heterogenous expression of Interferon γ (IFN-γ) was also detected in white adipose tissue (WAT). Differential expression of the pleiotropic cytokine, as well as the pathways it activates, could possibly be detected before the onset of refractory cachexia [51].

### 3.3. Interleukin 6 (IL-6)

Elevated serum levels of inflammatory markers such as IL-6 and CRP suggest inflammation as a common feature of cachexia and various inflammatory diseases (reviewed in: [52]).

Increased levels of cytokines, such as IL-6 in CC, may result in increased proteolysis and skeletal-muscle atrophy with preferential loss of myofibrillar protein, decreased food intake, and an increase in energy expenditure [27,53]. Furthermore, animal models were often used for the studies of the molecular mechanisms of CC, especially mice bearing the C26 carcinoma (also referred to as colon-26 (C26) adenocarcinoma) a model with high levels of IL-6 family ligands (reviewed in: [54]).

Pleiotropic effects of IL-6 action, including those that promote muscle atrophy, increased muscle wasting and the mechanisms of muscle wasting, are mainly described in animal models [4,53,55]. In mice affected by severe cachexia (Apc^Min/+^ mice), IL-6 overexpression altered the expression of proteins regulating mitochondrial biogenesis and fusion in cultured myoblasts (C2C12 cells). The affected specimen could be rescued by the administration of an IL-6 receptor antibody, as well as exercise [56]. Further studies proved that IL-6 overexpression in cachectic mice increases the levels of fasting insulin and triglycerides, with the possibility of their normalization through exercise, due to increased oxidative capacity, induction of Akt signaling, and down-regulation of AMP-activated protein-kinase (AMPK) signaling in muscles [57,58].

Another mouse-model study showed a phenotype switch from WAT to brown fat (WAT browning) during early CC stages, even before muscle atrophy. This occurs due to an increase in the expression of uncoupling protein 1 (UPC1) in WAT. This protein affects the mitochondrial respiration, uncoupling it toward thermogenesis instead of ATP synthesis, which results in an increase in lipid mobilization and energy spending in cachectic mice. In turn, the increase in UPC1 production in WAT is caused by the effects of IL-6, as well as the presence of chronic inflammation [59]. Similarly, Han et al. reported a correlation between chronic inflammation (especially that mediated by IL-6) and CC promotion. IL-6 concentration was correlated with serum-free fatty acids (FFA), both in early- and late-stage CC, while serum TNF-α was positively linked to FFA in early-stage but not late-stage CC. Furthermore, WAT lipolysis was increased in early- and late-stage CC, while WAT browning increased only in late-stage CC [60]. The WAT browning, as an effect of systemic inflammation, has a particular role in high energy expenditure associated with CC [59,60]. Recent studies showed augmented expression of pro-inflammatory cytokines, including IL-6, TNF-α, and IL-8 in CC patients as compared to the control group. Furthermore, IL-8 content was also higher in weight-stable cancer (WSC) patients compared to control. The plasma fatty-acid profile was also positively correlated with some of the pro-inflammatory cytokines expression in the CC patients (IL-8, IFN-γ, CCL2, and IL-1Ra) [61].

A comparison of serum concentrations of four pro-inflammatory factors (e.g., IL-6, IL-1β, Chemokine (C-X-C Motif) Ligand 8(CXCL8)/IL-8, and TNF-α) in advanced-stage cancer patients showed that IL-1β levels correlated more strongly with clinical characteristics than those of IL-6 [62]. There are also some clinical reports of resectable pancreatic cancer patients, which noted low levels of the described pro-inflammatory cytokines (IL-6, IL-1β, IFN-γ, and TNF-α), with their concentrations not correlated with CC, even if more sensitive methods were used for the analyses. Among the 25 tested circulating factors, only the monocyte chemoattractant protein 1 (MCP-1) showed an increase in treatment-naïve cachectic patients, as compared to those unaffected by the condition. Hence, it was suggested as a potential CC biomarker [63]. Recent studies by Cao et al. describe reporter cell lines that could detect factors associated with CC, such as myostatin, activin A, and TNF-α. This cell model could be a valuable tool of early-cachectic-state detection and differentiation of cancer-induced cachexia in humans and mice [64].

The works summarized above concern the correlations between cachexia determinants and SIR in cancers in general. It needs to be noted that a systemic increase in CRP and cytokines, e.g., IL-6, IL-1, and TNF-α is also observed with age (the so-called “inflamm-aging”), due to persistent sarcopenia. Furthermore, cohort studies mainly indicate TNF-α and IL-6 levels as cachexia markers (reviewed in: [65]).

## 4. Systemic Inflammatory Response in Colorectal Cancer-Associated Cachexia

A direct correlation between cancer cachexia and SIR was also confirmed in patients with CRC [40,66,67]. Most of the available literature concerns the role of the inflammatory process in muscle-tissue changes during CC. In turn, recent studies show a correlation between muscle catabolism and systemic inflammation in CRC. The presence of myopenia (reduced muscle mass) in preoperative CRC patients was significantly correlated with a number of SIR markers, such as elevated CRP concentration, systemic immune-inflammation index (SII), neutrophil-platelet score, and a decreased lymphocyte:monocyte ratio (LMR), prognostic nutritional index, and serum albumin level. Among those, only the increase in CRP level was indicated as an independent risk factor for the presence of preoperative myopenia [40]. Other reports indicate a correlation between elevated SIR, as measured by the high modified Glasgow prognostic score (mGPS), CRP, and neutrophil:lymphocyte ratio (NLR) with a low skeletal-muscle index in patients with primary operable CRC [68]. In turn, Malietzis et al. reported that host SIR in resectable CRC patients is associated with not only myopenia but also myosteatosis (increased infiltration by inter- and intra-muscular adipose tissue). High neutrophil:lymphocyte ratios (NLR) and low albumin levels were independent predictors of myopenia, with the former being correlated with myosteatosis [69]. Furthermore, Sirniö et al. noted correlations between serum levels of several amino acids (AAs) (low with glutamine and histidine and high with phenylalanine), SIR markers, and muscle catabolism in patients with CRC. Hence, the authors proposed several SIR indicators, e.g., mGPS, high blood NLR, and high serum levels of CRP, IL-6, and IL-8. Of the 13 cytokines tested, IL-6 most strongly associated with increased phenylalanine and lower histidine levels. The authors suggest that SIR is associated with glutamine consumption and muscle wasting in CRC [66]. In turn, the studies of Ohmori et al. suggest that only TNF-α, the levels of which were negatively correlated with skeletal-muscle index (SMI) and SDS-soluble myosin light chain 1 (SDS-MYL1), is a good serum muscle-atrophy marker in CRC-associated cachexia [67]. Other authors confirmed that higher TNF-α concentrations are usually associated with patients with more advanced CRC stages (stage III/IV vs. stage I/II tumors). In turn, patients with the greatest nutritional deficit exhibited higher levels of adipocytokines. However, these TNF-α variations were only significant in CRC vs. control when the nutritional status was evaluated by phase-angle tertiles [70].

There was also an attempt to evaluate the role of gut barrier disruption, possibly resulting in persistent activation of the host immune response, as a cause of local and systemic inflammatory changes in CRC-associated cachexia. Comparative studies concerned cachectic and weight-stable CRC patients, analyzing the circulating profile and tissue expression of various cytokines (including chemokines), growth and differentiation factors, as well as morphological intestinal changes in CRC-associated cachexia. Mucosal biopsies were derived from the rectosigmoid region, 20 cm from the tumor margin. An increase in serum concentrations of IL-6 and IL-8 was observed, together with elevated tissue expression of IL-7, IL-13, and transforming growth factor β3 (TGF-β3), as well as rich lymphocyte and macrophage infiltration in the colon of CC patients, compared to those of normal body weight. These changes suggest the presence of repair mechanisms in the damaged large intestine, with expanded recruitment of immune cells and higher tissue production of IL-13 and TGF-β3 [12].

Animal models also allowed for the clarification of some of the mechanisms of heart-muscle damage in CRC-associated cachexia involving inflammation. Studies on CD2F1 mice inoculated with C26 cells presented an increase in the production of IL-6, IL-6R, and F4/80 (a marker for macrophages infiltration) in the heart of tumor-bearing vs. non-tumor-bearing mice. Furthermore, increased fibrosis, disrupted myocardial structure, and altered composition of contractile proteins, e.g., troponin I (decreased), myosin heavy-chains isoforms α (decreased), and β (increased), was detected in the cardiac muscle of tumor-bearing mice, resulting in muscle-function impairment [71].

Inflammatory changes also play a role in metabolic disturbances observed in patients with CRC-associated cachexia. Mice bearing C26 carcinoma represented a well-established murine model of CC resulting in a high systemic level of IL-6 [72]. It was proven that these mice exhibit reduced adipose mass, increased AT lipolysis, and a five-fold increase in FFA plasma levels. These alterations were linked to the activation of IL-6 signaling in WAT through a three-fold increase in phosphorylated STAT3 and high suppressor of cytokine signaling 3 (SOCS3) gene expression levels [73]. It was also recently reported that the leukemia inhibitory factor (LIF) secreted by the tumor induces changes in the expression of AT, as well as serum levels of IL-6 and leptin, acting in a JAK-dependent manner. This, in turn, results in cachexia-associated adipose wasting as well as anorexia. The authors noted that the use of JAK inhibitors in both in vitro and in vivo models of CC inhibits this process [74].

Lipolysis is also a typical manifestation of CC. Some of the mechanisms of this process were elucidated, mainly in in vitro models, through the evaluation of the activity of enzymes involved in lipid degradation in cancer cachexia. One example of such is spermidine/Spermine N-1 acetyl transferase (SSAT), an important enzyme in polyamine metabolism, which plays other potential roles, e.g., decreasing lipid accumulation. Its activity is stimulated by a multitude of factors, including those associated with cachexia, such as several cytokines, hormones, and natural substances (reviewed in: [75]). Constant SSAT activation leads to an increased demand for acetyl-CoA (a cofactor of SSAT), thereby restricting conversion of acetyl-CoA by acetyl-CoA carboxylase (ACC) to malonyl-CoA [20]. Malonyl-CoA inhibits the rate-limiting step in the β-oxidation of fatty acids and is a substrate in fatty-acid synthesis. A study of the activity of enzymes responsible for increased lipolysis in a mouse model of cancer cachexia (C26) using a cachexigenic clone 20 (c20), and noncachexigenic clone 5 (c5), showed a significant increase in SSAT as well as a decrease in acetyl-CoA carboxylase (ACC) and malonyl-CoA in cachectic mice [76]. The studies of Chiba et al. confirmed these observations, demonstrating a correlation between malonyl-CoA in the liver and symptoms of CC (c5). In turn, in mice bearing the c5 tumor, the levels of SSAT mRNA did not correlate with the severity of CC manifestations. Hence, malonyl-CoA in the liver, correlating with the weight of fat stores in mice, seems to be a good marker of CC. A decrease in malonyl-CoA resulted in lowered fatty-acid synthesis and accelerated lipolysis via activation of carnitine palmitoyltransferase 1 (CPT-1) and ketogenesis. According to the authors, a decrease in the level of malonyl-CoA activity, playing a major role in CC induction, can be also caused by factors other than SSAT. From the panel of investigated pro-inflammatory (e.g., IL-1β, IL-6, and TNF-α) and anti-inflammatory/immunomodulatory cytokines (e.g., IL-10), only IL-10 had a negative correlation with body weight and the weights of skeletal muscle and storage fat [20].

In another study, Apc^Min/+^ mice (carrying a dominant mutation in the APC gene) are a model of colon cachexia directly related to an intestinal tumor burden and subsequent chronic inflammation (with elevated IL-6 levels). These mice were classified as noncachectic (8 weeks of age), precachectic (14 weeks of age), and severely cachectic (20 weeks of age). These studies confirmed the disturbances in hepatic triglyceride metabolism in catechetic mice. Furthermore, a novel role of hepatic glycosylphosphatidylinositol-anchored high-density lipoprotein binding protein 1 (GPIHBP1) was confirmed in hypertriglyceridemia with marked liver steatosis in Apc^Min/+^ mice. It was also reported that GPIHBP1 is involved in the NF-κB signaling pathway in the liver of cachectic mice. The authors found a decrease in the hepatic uptake of fatty acids and lipolysis, with no differences in fatty-acid β-oxidation [77].

Clinical studies of CC showed that the increase in serum FFAs (an indicator of enhanced lipolysis) was positively correlated with serum pro-inflammatory cytokine levels (e.g., IL-6 and TNF-α) [60]. In turn, disorders of lipid metabolism in cachexia (including mechanisms of lipolysis) were described in recent review papers. The involvement of both inflammatory factors, defects in energy utilization, and molecular mechanisms underlying the WAT dysfunction and browning in CC are all highlighted in the mentioned works [7,10].

The role of direct adipose-cell involvement in the mechanisms of lipolysis in cachexia is described later in the paper (Section 5.2). In turn, a brief summary of selected mediator action during systemic inflammatory response associated with CC is presented in Table 1.

## 5. The Role of Tumor Microenvironment (TME) Cells in CRC-Associated Cachexia

The tumor microenvironment (TME), apart from cancer cells (including CRC cells), mostly consists of blood vessels, various populations of stromal cells (e.g., fibroblasts, pericytes, and adipocytes), immune/inflammatory cells, ECM, secreted proteins, RNA, and small organelles [17,18,19,20,22,23]. In liver metastatic CRC, an analysis on the TME composition and characteristics identified a total of 12 clusters corresponding to six cell types, including cancer cells, T and B cells, myeloid cells, ECs, and fibroblasts [31]. TME resident cells partake in constant communication, mostly through the production and expression of cytokines (including chemokines) and growth factors, acting in autocrine, paracrine, and/or endocrine manner, all of which are critical to modulate the cellular and molecular events involved in maturation of the TME [19]. The differences in the phenotypic cellular composition of the TME, as well as its secretion ability in primary/metastatic CRC, can affect the progression and phenotype of cancer cachexia through alterations in various circulating factors.

### 5.1. Tumor-Infiltrating Immune Cells (TIICs)

This group of cells includes tumor infiltrating lymphocytes (TILs), composed of a mixture of adaptive immune cells (T and B cells), natural killer (NK) cells, macrophages and other innate cells (granulocyte, mast cells, and monocytes) in variable proportion, with T cells being the most abundant. After the determination of cell-surface antigens on lymphocytes (CD4+, CD8+, CD3+, etc.), it was recognized that the factors and/or cells that negatively regulate cytotoxic effects of TILs exist in the TME (reviewed in: [32]). The tumor-associated macrophages (TAMs) dominate among the immune/inflammatory cells of the TME in primary operable CRC, but lymphocytes and neutrophils can also be observed, with relatively few plasma cells or eosinophils [22,23,78].

#### 5.1.1. Tumor-Associated Macrophages (TAMs)

TAMs, classified as TIICs [79], are one of the most abundantly represented inflammatory cells in the TME of CRC (up to 50% of the tumor mass) [23,78,80,81]. They mostly occur in the form of M1 macrophages, serving a tumor-preventing role, and M2 macrophages with a tumor-promoting activity. A dual role of TAMs in CRC progression and development is often underlined [81,82,83]. Various phenotypic proportions of M1/M2 TAMs were described in CRC, ranging in correlations with clinical data, mostly concerning CRC patient survival. Ong et al. reported that TAMs were pro-inflammatory and inhibited the proliferation of tumor cells, as well as produced cytokines (e.g., IL-6 and IFN-γ) and chemokines (e.g., CXCL8/IL-8 and CCL2) that attract T cells, and promoted type-1 T-cell responses. Using CRC tissues, the authors confirmed that TAMs in vivo were pro-inflammatory and correlated with a number of tumor-infiltrating T cells. Hence, TAMs induced tumor-suppressive effects with the help of T cells [83]. Another study conducted on close to 500 CRC specimens showed a parallel growth of M1 and M2 infiltrations at the tumor front, with an inverse correlation of both phenotypes with tumor stage. No difference was detected in CSS in CRC groups with different M1/M2 (the nitric oxide synthase 2 (NOS2)^+^/CD163^+^, respectively) macrophage ratios. However, the studies suggest that the presence of numerous M1 macrophages is favorable for survival in patients with CRC, despite the presence of M2 macrophages [82]. The analysis of CD68^+^/iNOS-negative TAMs in CRC patients showed that the presence of intensive infiltrations of M2 macrophages, CD163^+^ [79] and CD68^+^ TAMs [84], in the tumor stroma was a bad prognostic, as it correlated with shorter DFS and OS. An association of CD68^+^ TAMs with relative risk of cancer recurrence was also reported, while the chance of cancer-related death was almost two times higher in CD68^+^/iNOS^−^ patients [84]. Similar correlation was presented for the numbers of stroma-infiltrating regulatory T cells (Tregs) [79,84]. These results were supported by bioinformatical studies. CRC patients with a lower abundance of CD163^+^ TAMs (M2) and Tregs had a longer DSF and OS, independent of chemotherapy. Considering the numbers of cells among the three TIIC subpopulations: CD66b^+^ TANs, FoxP3^+^ Tregs, and CD163^+^ TAMs, and based on ssGSEA and CIBERSORT tools, a new model of prognosis was created for use in effective prognostic evaluation of CRC patients [79]. Recent study results indicate the participation of CD163^+^ TAMs (M2) infiltrations in the invasive front of a tumor in EMT process, their association with mesenchymal-circulating tumor-cell (CTC) ratio, and poor prognosis in CRC patients. Through a production of IL-6, these cells regulated the EMT process, intensifying the migration and invasion of CRC cells. The main signaling pathway participating in this mechanism was the JAK2/STAT3/miR-506-3p/FoxQ1, which promoted macrophage recruitment through up-regulation of C-C motif chemokine ligand 2 (CCL2) secretion [85]. Some of the studies report that IL-6 produced by TAMs (M2) is also responsible for the induction of chemoresistance through the activation of IL-6R/STAT3/MiR-204-5p pathway in CRC cells [86]. Hence, the role of IL-6/JAK/STAT3 signaling in the TME and its possible application as a target in CRC therapy are currently considered [87].

The role of TAMs in CRC cachexia is not fully determined. Most of the results are derived from studies of mouse colon adenocarcinoma C26 model of cachexia, which since the 1990s suggest a role of IL-1 and IL-6 production-associated signaling pathways, as well as the intercellular interactions between IL-1R-expressing tumor cells and host-derived macrophages, hence the participation of the TME in CC [53,72,88]. An inductive influence of secreted cytokines and soluble factors from conditioned media of CRC cell lines (e.g., Caco-2) on differentiation of peripheral blood mononuclear cells (PBMCs), particularly monocytes, toward inflammatory phenotype. The treated monocytes showed increased amounts of IL-6, IL-12b, and IFN-γ and a decreased expression of IL-4, IL-10, and TNF-α [89]. In the C26 model of cachexia, a reduced number of macrophages and mesenchymal progenitor cells (as well as infiltrated neutrophils 24 h after muscle injury) was noted in cachectic mice. These cells are crucial in the first stages of muscle regeneration after injury. The regenerative ability of muscle stem cells was retained in vitro, but the proliferation and differentiation were severely impaired in cachectic mice in vivo. The study of CCL2–5 expression in muscle injured by cardiotoxin injection showed that the expressions of these chemokines were significantly reduced in C26-bearing mice compared to the control group (#KC group). In the mechanisms of disturbed skeletal-muscle regenerative ability in cachectic mice, a role of the chemokines representative for macrophage (and neutrophils) migration into damaged muscle is suggested. The C26 model studies primarily demonstrated a reduction in cell count (macrophages and neutrophils) and production of chemokines critical for skeletal-muscle regeneration. However, the proliferation and myogenic differentiation abilities of muscle satellite cells (MuSCs) were sustained in the cancer-cachexia muscle. The authors showed that damaged/necrotic myofibers occupied the largest area in the injured muscle and were smaller compared with non-transplanted and #KC groups, indicating that while muscle atrophy was induced in the C26-bearing mice, the muscle damage caused by cardiotoxin could not be compared with muscle damage during cachexia [90].

Among the other signaling pathways active in TAMs, the participation of the prostaglandin E2 receptor 2 subtype (PGE2/EP2) signaling is often underlined. PGE2 is the predominant prostaglandin involved in CRC pathogenesis. It is generated from arachidonic acid by cyclooxygenase-2 (COX-2) in an inflammatory-tissue condition, enhancing M2 macrophage polarization (reviewed in: [23]).

Other interesting mouse-model studies (AE17 mesothelioma-bearing female C57BL/6J mice during tumor growth) also demonstrate that age-related changes in macrophages are important drivers of CC in elderly down-regulating antitumor responses, particularly during immunotherapy. Reduction of such macrophages prevents this immunotherapy-induced cachexia [91]. However, similar studies were not conducted in CRC patients.

In summary, the role of TAMs in CRC-associated cachexia depends on the M1/M2 macrophage ratio in TME, as well as the immune response mediators produced by these cells (mainly IL-1 and IL-6). In CRC-associated cachexia, a cooperation between TAMs and cancer cells (mainly IL-1R-positive cells) can be observed, leading to phenotypic differentiation of monocytes toward the “pro-inflammatory” type (up-regulation of IL-6, IL-12b, and IFN-γ). These cytokines (mainly IL-6) produced by M2 cells can intensify tumor progression through regulation of the EMT process, as well as the migration and invasion of CRC cells. There are some opinions that the impairment of muscle regeneration in a mouse model of cachexia is caused by a decrease in the number of neutrophils, macrophages, and mesenchymal progenitors at the site of injury, which may be at least partly caused by a decrease in chemokine (CCL2-CCL5) production.

#### 5.1.2. Tumor Infiltrating Lymphocytes (TILs)

Tumor infiltrating lymphocytes composed of a mixture of adaptive immune cells (T and B cells) are also classified as TIICs, with T lymphocytes identified as a key component of this cell population [32]. Most of the results of studies on the particular TIL subpopulations presented in the “immune” tumor microenvironment have a prognostic value in CRC and serve for their more specific molecular classification and immunotherapy [68,78,92,93,94]. It was proven that TILs from MSI-H phenotype of CRC mostly contain lymphocytes with antitumor activity, including all of the CD4^+^ and CD8^+^ subpopulations, as well as activated dendritic cells (DCs) and NK cells. In turn, in microsatellite stable (MSS) CRC, the number of significantly depleted subpopulations was proportional to tumor heterogeneity [93]. A correlation was also reported between higher intratumoral heterogeneity and a greater mutational load in CRC, resulting in a higher load of neo-antigens and likely promoting T-cell activation and infiltration [94].

Considering the prognostic value of TILs in primary operable CRC, it was proven that lymphocyte and plasma-cell infiltration in the invasive margin of CRC are beneficial for survival [78]. Particularly strong infiltration of TILs (CD3^+^ at the invasive margin and CD8^+^ in the cancer cell niches) was correlated with improved CSS; hence, it could be important in predicting outcome in primary operable CRC patients [95]. Reissfelder et al. indicate TNF-α as a tissue marker of intratumoral cytotoxic T-lymphocyte activity. The authors reported that an up-regulation of TNF-α expression in TILs is strongly correlated with an increase in total intratumoral TNF-α [92]. It was also demonstrated that Th1 and cytotoxic CD8+-memory T-cell enrichment of the TME in primary CRC is associated with a reduced incidence of recurrence and/or metastasis. Furthermore, tumors with Treg and Th17 cells as TILs, as well as a molecular signature of CAFs, were associated with poor prognosis [32]. In turn, tissue studies of mesenteric lymph nodes (MLNs) and Peyer’s patches (PP) of CRC patients showed an increased number of CD8^+^/TCR^+^ and CD49b^+^/TCR^-^ cells (NK cells). Tumor tissues were also infiltrated by eosinophils, CD69^+^ T cells, and CD11b^+^ cells. Both in vivo tissues (CD45^+^ TILs) and cells of another murine CRC cell line (CT26), obtained from a BALB/c mouse, showed a positive reaction to IL-6, indicating this cytokine as key for CRC TME. Moreover, it could also potentially participate in CRC-associated cachexia [96].

In the CRC mouse model (CT26 and MC38 cells with an altered IL-6 expression), significant correlations between the expression of IL-6 and numbers of TILs in CRC were also demonstrated. The tumors with overexpression of IL-6 tended to grow faster than those with IL-6 knockout. In turn, a correlation between IL-6 overexpression and decreased number of CD8^+^ and CD4^+^ lymphocytes but increased myeloid-derived suppressor cells (MDSCs) and regulatory/suppressor T cells in the CRC tumor was also reported, with a similar connection established between the overexpression of programmed death-ligand 1 (PD-L1) (a potent inhibitory regulator of antitumor immunity) and IL-6 in CRC. These results indicate that IL-6 participates in immunosuppression induction in CRC TME through the recruitment of immunosuppressive cells and impairment of T-cell infiltration. In in vivo studies, a differential tissue expression of IL-6 was detected. Patients with higher levels of this cytokine in CRC tissues were characterized by a shorter overall survival (25.5 months average) compared to those with lower IL-6 expression (46 months) [97].

It is hard to comment on the role of TILs in CRC-associated cachexia, as there is a lack of studies focused on this topic. Shibata et al. showed a lowered expression of IL-12 and Th1 cytokines in PBMCs from patients with advancing CRC, with the lowest attributed to those affected by distant metastases and CC. In turn, the levels of Th2 cytokines (e.g., IL-4, IL-6, and IL-10) had increased in the same cachectic patients in terminal stage [98]. In mouse Apc^Min/+^ model, an altered level of CD8^+^ cells was described, together with changes in IFN-γ and granzyme-B expression by these cells. Furthermore, in MLNs and PP of these mice, lower levels of IFN-γ^+^/IL-17^+^ double-positive CD4^+^ cells were observed [99].

In summary, the research results indicate an imbalance of TIL (mainly T cells) cytokines/chemokines expression in various CRC models in vitro and in vivo, which is most likely involved in intestinal homeostasis impairment, resulting in a reduction in intestinal tumor immunosurveillance. Procachectic cytokine overexpression (IL-6, TNFα) in various TIL subpopulations and cooperation with CRC cells (including C26 cells) can result not only in CRC progression but also CC with TME cell population participation.

#### 5.1.3. Tumor-Associated Neutrophils (TANs)

TANs is an important population of TME immune cells, also classified in TIICs [32]. Similarly to TAMs, there are two subpopulations of these cells: N1—with antitumor phenotype, and N2—tumor promoting. The authors describe molecular signaling between TANs and CRC cells, their promoting role in CRC liver metastasis, as well as their potential targeting in antitumor therapy [22]. The heterogeneity of TANs, their plasticity, and a dual function in tumors are regulated by a number of TME factors and signals, mainly TGF-β and IFN-β signaling [22,100,101]. Moreover, the role of PGE2/EP2 signaling is also underlined in the promotion of CRC tumorigenesis [23]. EP2 stimulation in cultured neutrophils amplified the expression of TNF-α, IL-6, CXCL1, COX-2, and other pro-inflammatory genes synergistically with TNF-α [102]. Roles of these cells were confirmed during angiogenesis, tumor-cell migration, invasion, and metastasis [22,100,101]. Similarly to other TIICs, the prognostic role of TANs in CRC was also investigated [78,102,103]. Rao et al. observed an increase in the number of active (CD66b+) neutrophils in more than 45% of CRC patients and only in around 13% of adjacent mucosal tissues. Additionally, it was demonstrated that a decreased number of intratumoral neutrophils correlates with pathological metastasis/tumor size (pM/pT) status, clinical stage, and shortened survival in CRC patients, simultaneously being an independent factor of poor prognosis [103]. In turn, Governa et al. reported prevalent colocalization of TANs and CD8^+^ T cells in CRC, while functional studies determined that TANs enhance CD8^+^ T-cell activation, proliferation, and cytokine release. Additionally, CD8^+^ cell stimulation in the presence of CD66b^+^ neutrophils resulted in increasing numbers of “central memory” phenotype cells (CD45RO/CD62L). Better prognosis was associated with the presence of immune cell populations, namely CD66b^+^ and CD8^+^ T cells, rather than CD8^+^ T cells alone [104]. Similar results were obtained through a meta-analysis of almost 4000 solid tumors (including CRC). High levels of intratumoral TANs were associated with unfavorable recurrence-free survival, CSS and OS. In turn, peritumoral and stromal neutrophils were not significantly associated with survival. At the same time, this analysis suggests that CD66b+ cells might have a better prognostic value than CD15^+^ TANs [105]. This is also confirmed by the studies of Ye et al., based on bioinformatical methods and a predictive prognostic model. The results of which stated that CRC patients with a high infiltrating CD66b+ TANs density were associated with a better prognosis, as well as a longer DSF and OS, independent of chemotherapy [79].

A role of TANs is suggested in CC induction in colorectal cancer, based on the results of mouse-model studies. The analysis of the relationship between TME and CRC-associated cachexia using a C26 model (clone 5, c5) showed an increase in white blood cells (WBC), including neutrophils, which correlated with cachectic manifestation of mice bearing the c5 tumor. Neutrophil infiltration was also abundant in c5 tumors of mice inoculated in muscle tissue, peritoneal cavity, and thoracic cavity, all suffering from CC. Furthermore, the authors showed a correlation between the manifestation of CC and serum anti-inflammatory/immunomodulatory IL-10 levels but not other cytokines, among c5 groups, suggesting the involvement of similar mechanisms in cancer patients with CC [20]. These findings correspond with the previously published reports of the same group of authors, stating that the suppression of granulocyte differentiation by the enhancement of erythrocyte production results in the attenuation of CC manifestation [106]. In turn, the studies of Inaba et al., using FACS analysis, showed that apart from the reduction of macrophages and mesenchymal progenitors, the absolute number of infiltrated neutrophils in C26-bearing mice was lower than in control mice. The mechanisms of this process are still poorly understood, despite the reports of significant reduction or a tendency of a downregulation of expression of CCL2–5 and CXCL3 chemokines in C26-bearing mice. The CXC family of chemokines is important in the process of neutrophil attraction to the damage location. The authors discussed the potential mechanisms of muscle damage in CC, suggesting that the primary effect of CC was the impairment of neutrophil infiltration, resulting in disturbances in muscle regeneration [90].

In summary, rich intratumoral neutrophils infiltrations are a sign of bad prognosis in CRC patients. While the role of TANs in human CRC-associated cachexia is still unclear, it is known that these cells play an important role in CRC progression. In the tumor, TANs colocalize with CD8^+^ T cells, intensifying their activation and cytokine production. In the in vitro model, increased expression of procachectic cytokines/chemokines and other pro-inflammatory genes, synergistically with TNF-α, can be observed after EP2 stimulation. The involvement of neutrophils and critical chemokines in muscle regeneration has been suggested, but the exact molecular mechanisms require studies based on more accurate models of muscle injury in cancer cachexia.

#### 5.1.4. Myeloid-Derived Suppressor Cells (MDSCs)

MDSCs are a heterogenous population of immature myeloid cells in varying differentiation phases beyond the stage of myeloid progenitors, not including fully differentiated neutrophils, macrophages/monocytes or DCs, that exhibit a range of tumor promoting functions. These include stimulation of angiogenesis and tumor growth, as well as involvement in metastasis formation in cooperation with other cell types (reviewed in: [107]). Although not all MDSCs functions are fully understood, these cells are known to be metabolically active and immunosuppressive. They may play a role in inappropriate production of cytokines and inflammatory mediators that contribute to cachexia and sickness syndromes. Especially the expansion of metabolically active MDSCs, as immature granulocyte-differentiation antigen 1-positive (GR-1+) CD11b+ cells secreting large amounts of inflammatory cytokines (mainly TNF-α, IL-1, and IL-6), suggests a late development of the CC in the tumor-bearing host [108]. The TME also stimulates MDSCs by other factors induced by local hypoxia, namely hypoxia-inducible factors (HIFs 1 and 2) and low pH [109]. HIF-1α is associated with increased activity of arginase 1 (ARG1) and inducible-NO synthase (iNOS) in MDSCs, leading to a stronger inhibition of T-cell functions [110].

A role of MDSCs is suggested in CRC progression. A mouse model revealed that granulocytic MDSCs promote CRC cell stemness and progression in mice through exosomes enriched in S100A9 protein. More exosomes were formed under hypoxia and in a HIF-1α-dependent manner. Similarly, studies in CRC patients also show that human MDSCs enhance CRC cell stemness and growth via exosomal S100A9. Higher plasma exosomal S100A9 levels were observed in CRC patients vs. control [111].

In patients with CRC-associated cachexia, the role of MDSCs is combined with the chronic inflammatory process and the suppression of cell-mediated responses. MDSCs percentage in PBMCs increases particularly in terminal stages of CRC in patients with an impaired nutritional status observed in hypoproteinemia. Negative correlations have been shown between MDSCs level and total protein, as well as lymphocyte count, and positive correlation with neutrophil count and NLR [112]. Significantly elevated levels of circulating MDSCs positively correlated with sIL-2R levels, with the latter negatively correlating with nutritional parameters (e.g., total protein, pre-albumin, and transferrin), as well as the parameter of cell-mediated stimulation. Soluble IL-2R (sIL-2R) levels (similar to number of MDSCs) were directly proportional to neutrophil count and inversely proportional to the lymphocyte count [113]. Another study of gastrointestinal cancers (including CRC) demonstrated an inverse correlation between vascular endothelial growth factor (VEGF) concentration and the production of IL-12 and MDSC counts. Positive correlations have also been demonstrated between VEGF levels and neutrophil and neutrophil/lymphocytes counts, and negative correlation between the concentration of VEGF and lymphocyte counts. The results of these studies indicate that increased production of VEGF correlated with SIR, nutritional impairment and the inhibition of cell-mediated immunity involving MDSCs [114].

Summarizing the role of MDSCs in CC, these cells produce procachectic cytokines and chemokines, as well as interact with numerous mediators of chronic inflammation (e.g., IL-12, sIL-2R, and VEGF). Their growth was also influenced by other TME factors (e.g., HIFs, pH). MDSC levels (as a percentage of PBMCs) increase with the progression of nutritional disorders in CRC-associated cachexia. These cells are probably involved in immunological mechanisms (mainly inhibition of cell-mediated immunity) that induce CC in colorectal cancer.

### 5.2. Cancer-Associated Adipocytes (CAAs), Tumor-Resident Adipocytes (TRAs), Adipose Stromal Cells (ASCs)

In cancers that are located near the deposits of AT (including CRC), adipocytes undergo morphological and functional modifications due to their interaction with cancer cells (adipocyte dedifferentiation) and can be reprogrammed into cancer-associated adipocytes (CAAs), which are capable of producing pro-inflammatory agents [115]. Alterations have been detected in the levels of cytokines (e.g., adiponectin and ~11-fold increase in IL-6), as well as their tissue expression (e.g., five-fold increase in IL-6 in the subcutaneous AT (sAT)) in cachectic vs. weight-stable cancer patients [116]. Additionally, differential regulation has been observed in inflammatory and tumorigenic pathways in peritumoral AT (pAT) in CC. Numerous factors produced by the pAT, especially under inflammatory conditions (e.g., TNF-α, signal transducer and activator of transcription 1 (STAT1), and FAS-associated death domain (FADD)) were higher in CC than in the weight-stable CRC group. Furthermore, a number of mutual correlations were described between cytokines/chemokines produced in CC (e.g., positive between regulated on activation, normal T-cell expressed and secreted (RANTES) vs. IL-1R antagonist (IL-1Ra) and C-X-C motive chemokine 10 (IP-10) and negative with IFN-α)) [117]. All cytokines and growth factors produced and secreted by AT cells are important in the recruitment of various types of immune cells in TME, e.g., M1 and M2 macrophages. In the tumors of cachectic patients, a lowered amount of M2 cells was detected compared to weight-stable controls. In turn, in sAT of cachectic patients, a notably higher expression of TNF-α, IL-1β, and CCL2/MCP-1 genes, as well as several proteins (e.g., IL-1β, TNF-β, CCL3/MIP-1α, and CCL4) was described as compared to weight-stable patients [118].

Adiponectin is an interesting peptide hormone secreted by adipocytes, increasing lipid storage capacity within the adipose tissue, decreasing lipolysis, and enhancing lipid oxidation. It is also a key factor responsible for insulin sensitization. Epidemiological studies suggest an association between decreased serum levels of adiponectin and the risk of colorectal cancer. In contrast, the expression of adiponectin receptors (R1 and R2) shows much more variable expression in cancerous tissue (higher or lower compared to controls) (reviewed in: [119]). Unfortunately, a description of the exact function of this protein in the mechanisms of CRC-associated cachexia is still absent.

Other authors demonstrated augmented lipolysis in CC and major alterations in the expression of adipose triglyceride lipase (ATGL), hormone-sensitive lipase (HSL) (increase) and perilipin-1 (decrease) in the sAT of cachectic patients, as well as summarized the main changes associated with cachexia progression. In intermediate cachexia, an imbalance in the secretion of pro- and anti-inflammatory factors was observed. From a morphological point of view, a decrease in adipocyte size was demonstrated (early cachexia), together with profound changes in ECM and adipocyte shape (refractory cachexia) [120].

In gastrointestinal cancer cachexia, WAT architecture changes were also described, including an increase in fibrosis characteristics (accumulation of type I, II, and VI collagen, fibronectin, and elastic fibers) and inflammatory cell infiltration [29,30,121]. In turn, Batista et al. demonstrated the presence of macrophages and lymphocytes in AT especially in fibrotic areas, characterized by increased MCP-1 and CD68 gene expression in cancer patients. The researchers also noted reduced adipocyte size and AT atrophy [121]. Another study showed an increase in the content of α-smooth muscle actin (αSMA), and mRNA for fibroblast-specific protein 1 (FSP1, S100A4), particularly in the CC group. Hence, the presence of activated myofibroblasts surrounding adipocytes was confirmed, along with the increased of pro-inflammatory cytokine content (e.g., TNF-α, IL-6, and IL-8), with enhanced ECM protein synthesis due to cachexia. In the process of WAT fibrosis, TGF-β is often considered the most important factor. An increase was observed for the levels of both TGF-β1 and TGF-β3 in adipocytes, as well as downstream signals of TGF-β pathways (Smad3 and Smad4) in the fibrotic areas of cachectic AT [29]. Further studies confirmed higher tumor expression and plasma levels of IL-8, TGF-β1, and TGF-β3, as well as showed other factors’ increases (e.g., epidermal growth factor (EGF), GM-CSF, IFN-α, TGF-β2, and HIF-1α) in the tumor and serum of CRC cachectic patients. According to authors, tumor remodeling through TGF-β pathway activation in CRC-associated cachexia resulted in intratumoral inflammatory response, further causing the onset of fibrosis, transdifferentiation of TME fibroblasts to myofibroblasts, and an imbalance in inflammatory cytokine profile, all responsible for progression of the CRC [30].

There is a direct connection between AT cells (including adipocytes, macrophages, and other cells) and cancer-prone cells. As noted by excellent systematic reviews, interactions between these cells occur through the secretion of obesity-associated hormones (e.g., leptin, adiponectin, and IGF1), cytokines (including chemokines) (e.g., TNF-α, IL-6, IL-8, IL-10, and CCL2/MCP-1), VEGF and other activated CAA mediators. The main factors activating host cells of TME, acting in a para- or autocrine loop, include TNF-α, IL-6, and IL-8 [18,122,123]. These proteins activate cell-cycle regulators, as well as serve the roles of inflammatory/immune/angiogenic factors. Similarly, adipocyte-induced conditions (e.g., hypoxia) participate in the development and progression of various cancers [123]. Adipocytes localized in proximity of the invasive front of the tumor are reprogrammed by cancer cells into activated fibroblast-like cells. Active recruitment of adipocytes to the TME has not been reported, and the signals of their activation and recruitment by CRC cells are not yet known [18]. In other cancer types (e.g., prostate cancer) adipose stromal cells can migrate to tumors in response to the production of chemokines (CXCL1 and CXCL8) [124].

Among the chemokines produced, CXCL8/IL-8 secreted by a subpopulation of AT derived from stromal cells intensifies tumor invasion and growth of cancer cells via phosphatidylinositol 3 kinase (PI3K), Janus kinase/signal transducer and activator of transcription protein 3 (JAK/STAT3), as well as mitogen-activated protein kinase (MAPK) (originally called extracellular signal-regulated kinase (ERK) or the Ras-Raf-MEK-ERK) pathways [125]. In CRC cell lines, IL-6 influences the phosphorylation of MAPK/ERK, MEK1/MEK2, JAK2, and STAT3, the signaling pathways controlling cell metabolism and proliferation. On the mouse model of Apc/^Min/+^ leptin-induced IL-6 production was identified, together with STAT3 activation, as early events promoting proliferation of murine colon preneoplastic cells [126].

In conclusion, cancer-associated adipocytes produce numerous factors characteristic for inflammation, including typical procachectic cytokines (e.g., IL-6, TNF-α, and IL-1 β), chemokines (e.g., CCL2, CCL3, CCL4, CXCL8, and CXCL10/IP-10), and other proteins involved in multiple immune system functions (e.g., adiponectin, STAT1, FADD, and VEGF). These mediators play a key role in the recruitment of various types of immune cells (macrophages, lymphocytes) into the TME, as well as are responsible for interactions between CAAs and CRC-prone cells and the activation of myofibroblasts. TNF-α, IL-6, and IL-8/CXCL8 were identified as the most important factors in CRC induced cachexia involving CAAs.

### 5.3. Cancer-Associated Fibroblasts (CAFs)

CAFs are the most abundant component of non-cancerous cells in tumor stroma, playing a key role in the development of the TME. They participate in tumor growth, invasion, metastasis, cancer maintenance, and are attributed with a significant prognostic relevance. CAFs serve a dual inhibitory role in the context of immune cells, preventing their access to the TME and preventing their proper functioning within the tumor [127,128,129]. CAFs originate from different cell types including resident fibroblasts, bone marrow-derived mesenchymal cells, adipocytes, ECs, and stellate cells. The description of this heterogeneous group of cells, as well as their diverse functions in oncogenesis (from the promotion to inhibition of tumor development) are already a subject of a highly interesting review [130]. One of the characteristic elements of CAF activation in cancer is metabolic reprogramming. Various human tumors exhibit metabolic coupling between catabolic fibroblasts and anabolic cancer cells, mainly in the form of ROS-induced metabolic stress. The description of these dependencies, which may allow the explanation of some of CC’s pathomechanisms, were discussed in several existing reviews [127,131]. Authors of these works conclude that CAF catabolic processes result in the creation of the nutrient-rich microenvironment that metabolically supports tumor growth through local generation of mitochondrial fuels (lactate, ketone bodies, fatty acids, and glutamine, as well as other AAs) in the stroma [127].

In the case of CRC-induced cachexia, there are some noteworthy studies considering the subset of myofibroblastic CAFs in particular. This phenotype describes contractile cells that express αSMA and secrete ECM proteins, playing a critical role in tissue remodeling and fibrosis/desmoplasia [132,133]. Three characteristic factors for CRC progression include: transdifferentiation of fibroblasts to myofibroblasts, tissue remodeling through TGF-β pathway activation in cachectic CRC patients, and imbalance inflammatory cytokine profile [30]. The process of resident fibroblast activation to myofibroblasts involves the participation of tumor cells via the TNFR2/Akt/ERK signaling pathway, activated by progranulin, a protein secreted in the TME of CRC [134]. The pro-inflammatory signatures of myofibroblastic CAFs in colorectal cancer are composed of several cytokines (e.g., CCL2 and CCL8) which increase the migration and invasion of CRC cells (KM12 cell line) [132]. Production of IL-6 by myofibroblasts isolated from CRC was also noted, in an amount higher compared to tumor-cell secretion, inducing angiogenesis via the up-regulation of VEGF-A [135]. CRC cells can themselves increase the secretion of IL-6 from fibroblasts, which in turn induces invasion and expression of the integrin β6 adhesion molecule (an indicator of tumor progression) in CRC cells [136]. The presence of myofibroblasts surrounding adipocytes was also noted in CRC, along with the increased pro- (e.g., TNF-α, IL-6, and IL-8) and anti-inflammatory cytokine (e.g., IL-5) content, as well as enhanced ECM protein synthesis due to cachexia. The studies demonstrated that collagen deposition (mainly type I and III) was accompanied by excessive synthesis of mature elastic fibers, compromising the microenvironment of AT due to cachexia [29].

The desmoplastic signature of myofibroblastic CAFs includes 30 secreted proteins, some of them overexpressed specifically in the tumor stroma (e.g., follistatin-related protein 1 (FSTL1)). The combination of several biomarkers’ stromal expression (e.g., calumenin (CALU) and cadherin 11 (CDH11)) in CRC is linked with DFS and poor prognosis [132]. Furthermore, the molecular mechanisms of desmoplastic reaction in the CRC stroma, with the participation of CAFs expressing metalloproteinases (ADAMs), were recently reviewed by other authors [137].

Apart from the above-mentioned pathways (e.g., TGF-β and TNFR2/Akt/ERK), PGE2/EP signaling connected with stromal cells (including CAFs) also plays an important role in colon carcinogenesis [23]. The effects of this pathway’s activation in the context of cachexia include EP2 induction, the expression of which is in both neutrophils, and, CAFs in TME, which can result in regulation of inflammation- and growth-related gene expression in a self amplification manner. PGE2 released by CAFs in co-culture experiments suppressed NK-cell functions, in a newly described mechanism that links the pro-inflammatory and immunotolerance processes in CRC [138]. EP2 stimulation in cultured fibroblasts induced the expression of EP2, COX-2, IL-6, and Wnt genes. EP2 expression was also detected in clinical cases of ulcerative colitis-associated CRC. The authors suggest a promoting activity of CAFs and neutrophils via PGE2/EP signaling in colon carcinogenesis through the amplification of inflammation and TME shaping [102].

In conclusion, the role of CAFs in CRC-associated cachexia is particularly relevant to those of myofibroblasts phenotype, which play a role in tissue remodeling through enhanced ECM protein synthesis, fibrosis/desmoplasia, and an imbalance in the inflammatory cytokine profile. These cells produce numerous cytokines (including chemokines) (e.g., TNF-α, IL-6, IL-8, CCL2, and CCL8). Furthermore, activated CAFs are involved in the regulation of gene expression associated with inflammation and cancer growth and can combine pro-inflammatory effects with immunotolerance mechanisms. Overall, they promote tumor growth and can take part in CC.

### 5.4. Colorectal Cancer Cells

The role of CRC cells, especially in cooperation with other TME cells, is also crucial in cachexia. It is closely related to the secretion of procachectic factors, acting in an autocrine or paracrine manner, or transported to the blood and urine of patients. As early as two decades ago, it was first demonstrated that human CRC cells could be a source of the PIF glycoprotein [139]. Previous studies on mice adenocarcinoma of the colon 16 cells (MAC16 cells) demonstrated that this 24 kDa glycoprotein causes weight loss through the induction of enhanced protein degradation without a decrease in appetite. The protein induces catabolism of skeletal muscle [140], but also induces the production of cytokines (IL-6 and IL-8) in the liver, responsible for acute phase response, both resulting in unfavorable CC prognosis, accelerated weight loss, and shortened survival time [25,141]. PIF expression in CRC cells in vivo correlated with its presence in urine, as well as weight loss in cancer patients. Hence, this protein could also be used as a CC marker in patients affected with CRC [139,140]. In human CRC tissue, an overproduction of IL-6 and overexpression of IL-1β was reported, together with the expression of their proper receptors (IL-1R1 and IL-6R). The presence of these cytokines concerned both cancer epithelium and stromal cells. The co-expression of IL-1R1 and IL-6 was visualized in the same CRC cell line, determining the role of this cytokine network in CRC progression as crucial [142]. Hence, their participation is also possible in CRC-induced cachexia.

There are also some studies on the roles of cytokines and CC mechanism with participation of CRC tumor cells conducted on the mouse model of CRC-associated cachexia (C26 cells). The production of numerous factors by C26 cells important in the etiopathogenesis of cachexia has been demonstrated. IL-6 mRNA was detected at the tumor site of cachexigenic clone (c20)- but not at that of noncachexigenic clone (c5)-bearing mice. In contrast, higher TNF-α mRNA expression than in c20-bearing animals, both at tumor sites and in the spleens of c5-bearing mice, suggested that IL-6, not TNF-α, may be a key mediator of cachexia in this model. Thus, these studies demonstrated that IL-6 mRNA detection was limited to tumor sites in cachexigenic clone-bearing mice. However, continuous administration of mouse with recombinant IL-6 failed to induce cachexia. One the other hand, both C26 cell clones expressed IL-6 mRNA only in the presence of IL-1 in vitro, and mice bearing either of the clones expressed IL-1β mRNA at the tumor site. These authors also demonstrated the regulation of IL-6 expression through IL-1β and IL-1Ra. Overall, the study also showed that IL-6 is necessary but not sufficient for the induction of cachexia. Also, other cachexigenic factors besides IL-1Ra and IL-1β may control IL-6 production and induce cachexia in this model [143]. A positive reaction to IL-6 was also demonstrated in a murine CRC cell line (CT26) derived from a BALB/c mouse [96]. IL-6 expressed by tumor cells, both in mouse models and in CRC patients, is critical in modeling immune responses in cancer, as well as inducing strong immunosuppression in the CRC microenvironment [97,144]. Toyoshima et al. showed that IL-6 promotes metastatic colonization of C26 colon cancer cells, as well as dysfunctional antitumor immunity [144]. In turn, Li et al. observed increased expression of IL-6 in close to 60% of CRC patients, which tended to have shorter survival than the patients with low IL-6. In mouse CRC model, tumors with IL-6 overexpression tended to grow faster than IL-6 knockouts. Moreover, overexpression of IL-6 was correlated with impaired T cell infiltration (decreased number of CD8^+^ and CD4^+^ lymphocytes), as well as recruitment of immunosuppressive cells (more numerous MDSCs and regulatory/suppressor T cells) [97]. In turn, TNF-α-initiated inflammatory signaling in CT26 cells selectively induced PD-L1 expression in mesenchymal stromal cells, leading to down-regulation of CD8^+^ T-cell proliferation, as well as activation and promotion of CRC growth in vivo [145]. The role of Fn14, a cognate receptor of the cytokine TNF-like weak inducer of apoptosis (TWEAK) of the TNF/TNFR superfamily, was also indicated in CC initiation, as its overexpression was confirmed in C26 cells. In several solid tumors (including CRC), positive correlation was demonstrated between Fn14 and IL-1α and β, IL-6, IL-8, and TNF-α expression [146]. It was also reported that an increase in C26-tumor-derived serum levels of S100B and high mobility group box 1 (HMGB1) leads to persistent activation and overexpression of the receptor for advanced glycation end-products (RAGE), a protein-inducing key-changes characteristic for CC (e.g., muscle wasting, systemic inflammation, and release of tumor-derived procachectic factors) [147]. Similarly, C26 cell studies identified tumor-secreted factors (called “cachexokines”), e.g., bridging integrator 1, syntaxin 7, multiple inositol-polyphosphate 1, glucosidase alpha acid, chemokine ligand 2, Adamts like 4, and ataxin-10, which induce cardiomyocyte atrophy and aberrant lipid metabolism. Among those factors, ataxin-10 was determined as a prototype marker of cardiac cachexia in mouse and human CC models [148]. Other experimental studies employing the C26 model showed that IL-6 and associated cytokines are responsible for muscle wasting in clinical and experimental CC, as well as that this action occurs via JAK/STAT3 signaling. The main mediator of muscle wasting in cancer cachexia is STAT3, together with a range of conditions associated with high activity of the IL-6-family signaling [53]. In the same model of cachexia studies, it was proven that media derived from C26 tumor cells are able to induce C2C12 myotube atrophy and activate a STAT-dependent reporter gene in myotubes. In turn, among the several investigated gp130 family members, this effect was mediated by two factors: oncostatin M and LIF (and not IL-6 or TNF-α), as well as the activation of LIF/JAK2/STAT3 signaling [149]. The same research group demonstrated that LIF is the dominating cachexia initiating factor for C26 tumors in vivo. Furthermore, it was proven that increases in the levels of IL-6 and G-CSF are dependent on tumor-derived LIF [150]. As it was mentioned, it was also reported that Caco-2 cell-conditioned media caused the differentiation of PBMCs, particularly monocytes, toward inflammatory phenotypes [89]. The effects of altered cancer-cell metabolism, as well as detailed aspects of metabolic crosstalk between CRC cancer cells and TME cells, were reviewed recently in excellent publication. These interactions result in T cells causing immunosuppression, metabolic reprogramming of CAFs toward glycolysis, and aberrant function of ECs augmenting the glycolysis process [151].

In conclusion, the role of CRC cells is related to the production of classic cachexia-inducing factors (e.g., PIF, IL-6 and IL-6R, TNF-α, IL-1β, IL-1R1, and LIF), as well as novel factors known as “cachexokines”. The first procachectic factors mentioned are mainly responsible for increasing skeletal-muscle catabolism, accelerating weight loss and shortening survival time. CRC cell product (mainly IL-6) also mediate the recruitment of immunosuppressive cells (e.g., MDSCs and Treg) and reduce T-cell infiltration. This results mainly in cell-mediated response suppression and CRC progression. A group of these cachexokines (with ataxin-10 as a prototype marker) is responsible for specific cachectic changes in the heart muscle (cardiomyocyte atrophy and altered lipid metabolism).

The role of TME cells as well as potential associated mechanisms of CRC induced cachexia is presented in Table 2.

## 6. The Main Signaling Pathways Involved in Skeletal-Muscle and Adipose-Tissue Alterations in Cancer Cachexia

### 6.1. Skeletal Muscle

Studies of the mechanisms of skeletal-muscle atrophy in CC indicate the presence of various forms of damage but primarily disruption of protein synthesis, increased protein degradation, or both [7,25,26]. Abnormalities in skeletal-muscle regeneration in CC have also been described. Satellite cells are able to proliferate and can be activated, but they cannot complete their differentiation process into skeletal-muscle cells, due to persistent expression of paired box 7 (PAX7) via NF-κB activation [7]. Both IL-1 isoforms (α and β), through an oxidant and Akt/FOXO-independent mechanism and p38 MAPK activation, stimulate the nuclear factor kappa B (NF-κB) signaling. This led to skeletal-muscle cells differentiation disorders and myotubes catabolism [50]. TNF-α and PIF have a direct effect on skeletal muscle, through the activation of NF-κB signaling and induction of ubiquitin-mediated proteasome degradation of muscle protein [5,42,44]. The main molecular pathways that mediate muscle wasting and CC in pancreatic cancer include TGF-β, myostatin and activin, as well as IGF-1/PI3K/Akt and JAK/STAT3 signaling [152,153]. The details on the connections between STAT3 signaling (especially as a mediator of IL-6 and related proteins) and multi-organ manifestation of CC are already reviewed by others [154].

In summary, the main mediators of skeletal-muscle wasting in CC include cytokines (e.g., IL-1, TNF-α, and TGF-β), myostatin, and activin, as well as various intracellular signals activated by PIF. Cytokines and PIF act through NF-κB stimulation, with FOXO activation leading to increased transcription of Atrogin 1 and MURF1, which in turn promotes muscle-protein degradation. The activation of p38 and Janus kinase (JAK)/MAPK cascades by PIF, cytokines and myostatin, results in apoptosis. Myostatin can activate protein degradation through FOXOs and decreases protein synthesis through inhibition of Akt via Smad. Another type of signaling inhibiting protein synthesis is the Insulin-like growth factor 1 (IGF1) pathway. Decreased IGF1 during CC plays protein synthesis inhibiting role in cachexia. In turn, the peroxisome proliferator-activated receptor-γ co-activator 1α (PGC1α) pathway increases UPC expression that damages mitochondrial function (reviewed in: [7]).

In the case of CRC, mouse-model studies of Zhou et al. demonstrated that cachexia induced by murine C26 cells may be partially attributed to the enhanced TNF-α and IL-6 levels, which are controlled by the NF-κB signaling [155]. The use of various cancer-induced cachexia in vitro models allowed the demonstration of a key involvement of IL-6/STAT3 signaling in the process of skeletal-muscle wasting [6,53,156]. The C26 model also indicated a role of LIF/JAK2/STAT3 signaling in myotube atrophy in CC [149]. In murine models of cachexia (C26 and Lewis lung carcinoma cells), it was proven that the S100B protein (a ligand of RAGE) is able to induce muscle atrophy through the activation of p38 MAPK/myogenin/atrogin-1 axis and STAT3-dependent MyoD degradation [147].

A number of works reviewed by Hardee et al. that concern the cancer-induced metabolic and energy stress in skeletal muscle, indicate persistent increase in muscle AMPK activity during late-stage cachexia, coinciding with the suppression of mammalian target of rapamycin complex 1 (mTORC1) signaling and disrupted mitochondrial function [58].

### 6.2. Adipose Tissue

In addition to changes in muscle tissue, impaired adipose-tissue function is also observed in CC [7,26]. Loss of AT is associated with increased lipolysis and may also result from the inhibition of adipogenesis and/or reduced lipogenesis. Major lipolysis is promoted by lipase activation, zinc-α2-glycoprotein (ZAG) and pro-inflammatory cytokines (e.g., IL-1, IL-6, and TNF-α), resulting in high levels of circulating FFA and glycerol. The browning of WAT observed in CC can be also promoted by cytokines, ZAG (a lipid mobilizing factor), and tumor-derived factors, e.g., parathyroid-hormone-related protein (PTHRP) [7,26,59,60].

Most of the intracellular signaling pathways associated with TNF-α have been described in non-adipose cells. The best-known mechanism of TNF-α action on adipose tissue is the stimulation of lipolysis. Its activation leads to phosphorylation and down-regulation of perilipin via the MAPKs p44⁄42 and JNK, but not p38. In human adipocytes, this cytokine acts exclusively through TNFR1. In animal (rats) cells, TNF- α down-regulates the expression of the Gα_i_ antilipolytic GTP-binding membrane proteins. Unfortunately, the mechanisms of action of this cytokine in alteration of AT in CC are still very poorly understood (reviewed in: [157]). Considering other signaling pathways in AT, TGF-β pathway was a key signaling component in WAT wasting and induction of WAT fibrosis in cachectic patients with gastrointestinal cancers (including CRC) [29]. Moreover, in CRC-associated cachexia, activation of TGF-β signaling was also demonstrated, contributing to the onset of fibrosis in the tumor via non-canonical MAPK pathway [30].

## 7. Autophagy and Cancer Cachexia—Role of TME Cells

Not all mechanisms of tissue damage in CC are clear, with autophagy being increasingly considered in the pathomechanisms of CC in general and in muscular atrophy in particular [3,42,158,159].

Autophagy is the naturally regulated mechanism of the cell that removes unnecessary or dysfunctional components through autophagic-lysosomal proteolysis. In physiology, the role of autophagy includes the maintenance of muscle mass, adipogenesis, regulation of cytokine production and immune cell-B and -T development. Its general functions include starvation-induced AAs production, turnover of cytoplasmic contents, selective degradation of p62, impaired mitochondria, and anti-aging activity [160].

In C26 model of cachexia, the stimulation of muscle autophagy exacerbated muscle atrophy in tumor-bearing mice. The excessive autophagy induced in the muscle of these mice, together with an increase in UPS activation, resulted in degradation of both proteins and mitochondria. Excessive autophagy might impair mitochondrial function through the so called mitophagy. The authors showed that muscle-specific autophagy induction via TP53INP2 overexpression exacerbates cachexia [159]. Other studies provided direct evidence for p38α/β MAPK signaling in meditation of oxidative stress-induced autophagy-related genes, suggesting that this signaling pathway regulates both the UPS and the autophagy systems in muscle wasting [161]. It was also observed that cardiac muscle mass loss in the Apc^Min/+^ mice was associated with the Akt-independent suppression of anabolic signaling and increased autophagy [162]. Very interesting results indicate that human CC leads to exacerbated muscle-stress response, resulting in muscle loss, partly due to disruption of mitochondrial morphology, impaired autophagy, and increased apoptosis [163].

Autophagy may also play an important role in the crosstalk between tumor and stromal cells of TME. The process becomes more important for cancer-cell survival once tumors are established [160]. In advanced stages of cancer, autophagy promotes the survival of tumor cells by ameliorating stress in the TME (reviewed in: [164]).

Tumor stromal autophagy can also support tumor development by providing tumor-promoting factors and stimulating immunosuppression (reviewed in: [165]). As mentioned, when describing the role of CAFs in cachexia, it has been suggested that the catabolic phenotype of CAFs results from the activation of HIF-1α and NF-κB signaling that lead, i.e., to autophagy in stromal fibroblasts [127]. In turn, oxidative stress in fibroblasts induces autophagic destruction of mitochondria by mitophagy. Fibroblasts are then forced to undergo aerobic glycolysis and produce energy-rich nutrients (e.g., lactate and ketones) to reutilize their own constituents for energy balance and to “feed” cancer cells. The authors called this phenomenon “the autophagic tumor stroma model of cancer metabolism” or “reverse Warburg effect” [166,167].

**Figure 1:**
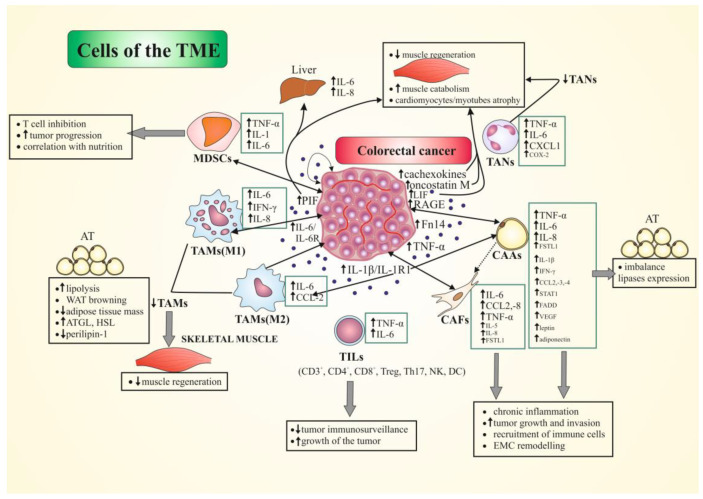
Schematic illustration of the tumor microenvironment (TME) cells involved in the development of colorectal cancer (CRC) cachexia through the secretion of specific products. [↑, ↓—increase/decrease; AT—adipose tissue; ATGL—adipose triglyceride lipase; CAAs—cancer-associated adipocytes; CAFs—cancer-associated fibroblasts; CCL2,- 3, -4, -8—the chemokine (C-C motif) ligand 2/3/4/8; CD3^+^—cluster of differentiation 3^+^, etc.; COX-2—cyclooxygenase-2; CXCL8—the chemokine (C-C motif) ligand 8/IL-8; FADD- FAS-associated death domain; Fn14—the cognate receptor of the TWEAK of the TNF/TNFR superfamily; FSTL1—follistatin-related protein 1; HSL—hormone-sensitive lipase; IFN-γ—interferon gamma; (IL-) 6R—(interleukin) 6 receptor; LIF—leukemia inhibitory factor; M1/M2—two phenotypes of macrophages; MDSCs—myeloid-derived suppressor cells; PIF—proteolysis-inducing factor; RAGE—receptor for advanced glycation end-products; STAT1—signal transducer and activator of transcription protein 1; TAMs—tumor-associated macrophages; TANs—tumor-associated neutrophils; TGF-β—tumor growth factor beta; Th17—T-helper cells 17; TILs—tumor-infiltrating lymphocytes; TNF-α—tumor necrosis factor α; Treg—T-regulatory cells; TWEAK—TNF-like weak inducer of apoptosis; VEGF—vascular endothelial growth factor].

The tumor microenvironment cells involved in the development of cancer cachexia in CRC are presented on Figure 1.

Within the TME, there is an array of resident tumor-associated immune and non-immune cells contributing to colorectal cancer progression and, consequently, also in CRC-associated cachexia. Their secretory products, e.g., cytokines (including chemokines), hormones, growth, and differentiation factors, are presented. The main targets of CRC-associated cachexia are the adipose tissue (AT), as well as skeletal and cardiac muscle. Arrows indicate positive regulation.

## 8. Therapeutical Options for Cancer Cachexia in CRC

Modern therapeutic strategies in CRC take into account not only metabolic changes in tumor cells [151] but also TME components [19]. Thus, therapeutic targets are increasingly TME cells, e.g., macrophages and MDSCs (Pexidartinib, PLX3397), as well as cytokines, e.g., IL-1/IL-1R (Canakinumab, Ilaris). However, such therapies are mainly at the stage of preclinical studies. Occasionally, they are based on complex treatment protocols, including, among others, dual metabolic inhibitors in combination with chemotherapy and immunotherapy [19].

Although effective pharmacological therapy for patients with cancer cachexia is still lacking, anticachectic treatment usually includes several strategies, primarily with anti-inflammatory/anticytokine agents and improving the patient’s nutritional status [168,169,170,171]. Some positive anticachectic effects (e.g., alleviated tumor-free body-weight reduction) have also been demonstrated in the mouse model using combination therapies, e.g., GH + insulin + indomethacin [172]. Anti-inflammatory treatment involves blocking the action of cytokines with a key role in the pathogenesis of CC, such as TNF-α, IL-1, IL-6, and INF-γ [168,169]. As early as 10 years ago, relatively new anticachectic agents included Tocilizumab (anti-IL-6 receptor mAb), IL-1 receptor antagonist (IP 1510), or selective androgen receptor modulators (Enobosarm and LGD-4033). Other candidates included the anabolic/catabolic transforming agent MT-102, naturally occurring peptide hormones, such as ghrelin and active ghrelin receptor agonist (anamorelin), or ARA284 and ARA286 erythropoietin analogues [170]. In later years, it was shown that therapeutic agents such as those already mentioned (Tocilizumab, Enobosarm, and Enamorelin), Infliximab (chimeric anti-TNF-α MAbs), MABp1 (for IL-1α blockade) or bimagrumab (BYM338, human monoclonal antibody to treat muscle loss) showed significant impact on reversal of skeletal-muscle loss, with limited effect on physical function [173].

There are drugs used in pancreatic cancer-induced cachexia that inhibit cytokines such as e.g., TGF-β, TNF-α and IL-6. A drug called Trabedersen binds TGF-β mRNA. In contrast, TNF-α is blocked by Infliximab and Etanercept (recombinant TNF-αR), as well as thalidomide/lenalidomide and pentoxifylline. In turn, IL-6 activity is inhibited by drugs such as the aforementioned Tocilizumab and Clazakizumab (anti-IL-6 mAb). In addition, innovative nutritional strategies for cachexia management in this type of cancer include ketogenic diet, utilization of natural compounds such as silibinin, and supplementation with ω3-polyunsaturated fatty acids (reviewed in: [152]).

A discussion of available anticytokine therapies for CC caused by various types of cancer (including CRC) is available in a recent excellent review [171]. The authors note phase I and II clinical trials using anticytokine therapy for the treatment of CC, and in some cases positive results in phase III trials as well. The most promising anticachectic agents (also in CRC cachexia) are MABp1 [174,175] and Thalidomide [171,176].

Improving nutritional and metabolic parameters in incurable cancer, as well as in CC, has also been a leading topic in the recent years [177,178,179]. According to some recent reviews “nutritional intervention is recommended as a cornerstone of multimodal therapy” in cancer cachexia (reviewed in: [180]). In the mechanisms of anorexia in cancer, orexigenic peptide, agouti-related protein (AgRP) and ghrelin have been observed to decrease pro-inflammatory cytokines and to increase body weight, food intake, and muscle mass in CC [177]. Recent studies indicate that the ghrelin-receptor agonist (anamorelin) is also effective in CRC-induced cachexia. This drug was approved for CC therapy in Japan in December 2020 [181,182].

Other therapeutic approaches to reduce the effects of cachexia, tested so far in a mouse model, offer hope for their use in humans as well. These include antibodies against Fn14 [146] and factors against RAGE [147]. The absence of this protein in mice resulted in reduced serum levels of cachexia-inducing factors, delayed loss of muscle mass, and strength, reduced tumor progression, and increased survival [147]. Furthermore, an inhibitory and slowing effect on CRC progression has been attributed to a combination therapy using a neutralizing antibody against activin receptor type 2 (anti-ActRII) called CDD866, in combination with cisplatin or everolimus [183]. Reduction of CC by a mechanism of lowering pro-inflammatory cytokines in a mouse model of C26 adenocarcinoma has also been shown after treatment with the *Citrus unshiu* plant peel extract (CUPE) [184].

A summary of therapeutics used or promising for the treatment of CRC induced cachexia is presented in Table 3.

## 9. Concluding Remarks and Future Perspectives

Cancer cachexia is a multifactorial paraneoplastic syndrome characterized by wasting of adipose tissue, as well as skeletal and cardiac muscles. Cytokines, including TNF-α, interleukins-1, -6, and interferon-α and -γ are known mediators of the cachectic process. These cytokines are produced by the host cells in response to the tumor, as well as by the tumor cells themselves. Cachexia, as a complication of tumor growth and progression, results in significant changes in host metabolism. Due to a lack of effective methods of treating CC, a significant factor affecting patient life quality and prognosis, it is necessary to fully elucidate the molecular mechanisms of this syndrome. In colorectal cancer, cachexia affects around 50–61% of patients and is the cause of death in at least 20%.

The model of mice bearing the colon 26 (C26) adenocarcinoma was particularly useful for the analysis of mechanisms involving tumor microenvironment cells in CRC. Using this model, a group of tumor-secreted factors called cachexokines (e.g., ataxin-10) were identified, together with other new proteins inducing key changes characteristic for CC (muscle wasting, systemic inflammation, and release of tumor-derived procachectic factors, e.g., RAGE).

The results point to complex tumor–host interaction that directly or indirectly lead to the destruction of muscle and fat tissue. The activity of all cellular components of the TME is also affected by other factors of the tumor environment (e.g., pH and hypoxia).

The presence of different phenotypes of TME cells and, in the case of particular immune cells (e.g., macrophages and neutrophils), also their number have prognostic significance in CRC. Most TME cells in CRC interact with each other and with tumor cells during the production of procachectic factors. These include mainly pro-inflammatory cytokines (e.g., IL-1, IL-6, and TNF-α), some chemokines (e.g., IL-8), and hormones (e.g., angiotensin II, leptin, glucocorticoids, and IGF1), and other factors (e.g., PIF, LIF, oncostatin M, and myostatin). Among these factors, pro-inflammatory cytokines (e.g., IL-1, IL-6, and TNF-α), myostatin, and PIF are the main contributors to the muscle wasting syndrome. Furthermore, the same pro-inflammatory cytokines (e.g., IL-1, IL-6, and TNF-α) are involved in adipose-tissue damage in CC.

It should also be noted that virtually all TME cell populations can be the source of procachectic factors, with their action also dependent on their proportion in the site of action. However, the overlap of signaling pathways characteristic for different stages of tumor progression and cachexia makes it difficult to interpret the role of individual mediators of this process. The mechanisms of procachectic action of TME cell products in CC demonstrated in different CRC models indicate their high complexity. Furthermore, not all mechanisms of cachexia involving TME cells can explain their clinical manifestations in humans with cancer (including CRC).

In conclusion, assessing the role of single TME cells in CRC-associated cachexia is difficult. Numerous procachectic mediators/factors/biomarkers are produced by tumor cells, tumor-infiltrating cells, as well as peripheral tissue parenchymal cells and associated infiltrating cells. The secretion products of all TME cells affect other organs and systems (tumor macroenvironment). Due to the lack of an ideal CRC induced in vivo cachexia model, as well as overlapping mechanisms and signaling pathways in CRC progression and cancer-induced cachexia, the role of TME cells in these processes is very difficult to analyze.

On the basis of the literature review, however, it can be confirmed that the decisive role of pro-inflammatory cytokines (e.g., TNF-α, IL-6, and IL-1) and certain chemokines (e.g., CXCL8/IL-8), as procachectic agents produced by many types of TME cells, is also relevant in CRC-associated cachexia. Further research is required to elucidate the detailed mechanisms of malnutrition in CC or autophagy processes associated with TME cells in the induction of human cancer cachexia. In addition, the development of better experimental models could allow for more accurate study of the interaction of the TME with tumor macroenvironment (organs, systems), allowing one to delay the development of cachexia and/or design more effective methods of its therapy. There is an urgent need for the development of effective TME biomarkers as therapeutic target in the context of CRC induced cachexia.

## Figures and Tables

**Table 1:** Summary of some mechanisms and effects of selected procachectic mediators involved in the cancer cachexia.

Procachectic Mediator	Model of the Study	Target Tissue	Mechanism of Action	Ref.
TNF-α	Murine C2C12 and primary myoblasts; mouse colon-26 cells (C26); various in vitro and in vivo mouse models of CC; patients with CC	Muscle	(i) catabolic effects; (ii) TNF-α + IFN-γ led to ↓expression of myosin via RNA-dependent mechanism in myotubes and muscle tissue; (iii) UPS pathway contribution	[44]
		Adipose; muscle	(i) catabolic effects; (ii) loss of AT and proteolysis, while causing ↓protein, lipid, and glycogen synthesis; (iii) ↓lipoprotein lipase; ↑gluconeogenesis; (iv) ↓protein synthesis via ↓in the active eIF4F complex; (v) serum levels do not correlate with weight loss	[5,41]
	CRC patients with different stages of CC; BALB/c male mice; C26/clone 20 cells	Adipose	was positively linked to FFA in early- but not late-stage CC	[60]
	cancer patients with CC; WSC; C		↑level in CC patients vs. C	[61]
	CRC tissue samples	Muscle	(i) negative correlation with SMI and SDS-MYL1; (ii) positive correlation with HMGB1	[67] *
	primary operable CRC and control		(i) ↑level was correlated with CRC staging; (ii) patients with the greatest nutritional deficit exhibited ↑levels of adipocytokines	[70] *
PIF	various mouse models of CC; cancer patients with CC	Muscle	(i) catabolic effect on skeletal muscle; (ii) ↓protein synthesis via ↑phosphorylation of the eIF2 on the alpha-subunit; (iii) its presence is indicative of weight loss; (iv) its presence in the urine of CC patients is indicative of weight loss	[41]
	C2C12 mouse myoblasts; MAC16 tumors	Muscle	↑Ca^+2^_i_, initiating a signaling cascade that leads to a ↓protein synthesis and ↑ in protein degradation	[45]
IL-1 (α and β)	C2C12 myoblasts, mature C2C12 myotubes model	Muscle	(i) an oxidant and Akt/FOXO-independent mechanism to activate p38 MAPK, ↑NF-κB signaling; (ii) ↑expression of Atrogin 1/MAFbx and MuRF1, and ↓myofibrillar protein in differentiated myotubes; (iii) the direct mechanisms in human CC tissue wasting are not recognized	[50]
IFN-γ	Various in vitro and in vivo mouse models of CC	Adipose; muscle	(i) with TNF-α led to ↓expression of myosin via RNA-dependent mechanism in myotubes and muscle tissue; (ii) UPS pathway contribution; (iii) changes in expression and signaling may be perceived at stages preceding refractory cachexia	[44,51]
	cancer patients with CC; WSC; C		positive correlation with plasma FA profile	[61]
IL-6	Apc^Min/+^ mice; C2C12 cells	Muscle	(i) ↑muscle wasting; (ii) altered the expression of proteins regulating mitochondrial biogenesis and fusion; (iii) ↓in mitochondrial content during the progression of cachexia; (iv) directly ↑FIS1 expression in muscle cells; (v) ↑indices of ROS in myotubes	[56]
	mature C2C12 myotubes model; in vitro and in vivo mouse model of CC	Muscle	STAT3 activation is a common feature of muscle wasting, muscle fiber atrophy and exacerbated wasting in CC	[53]
	Mouse model of CC; C26 cells; human cancerous-tissue samples	Adipose	↑expression of UPC1 in WAT, which affects the mitochondrial respiration, uncoupling it toward thermogenesis instead of ATP synthesis, which results in ↑lipid mobilization and energy spending in CC	[59]
	CRC patients with different stages of CC; BALB/c male mice; C26/clone 20 cells	Adipose	(i) regulating WAT lipolysis in early-stage cachexia and browning in late-stage cachexia; (ii) correlation with serum FFA	[60]
	cancer patients with CC; WSC; C		↑level in CC patients vs. control	[61]
	C26 model of CC	Adipose	(i) ↓AT mass, ↑AT lipolysis, and a 5-fold ↑ in FFA plasma levels; (ii) activation of IL-6 signaling in WAT through a 3-fold ↑ in pSTAT3 and high SOCS3 gene expression levels	[73] *
	CD2F1 mice inoculated with C26 cells and vehicle	Muscle	(i) ↑IL-6, IL-6R, and F4/80 in the heart of tumor-bearing vs. control; (ii) ↑fibrosis, disrupted myocardial structure, and altered composition of contractile proteins	[71] *
IL-8	cancer patients with CC; WSC; C		(i) ↑level in CC patients vs. control and in WSC vs. control; (ii) positive correlation with plasma FA profile	[61]

**Legend:** ↑, ↓—increase (up-regulation)/decrease expression/level; *—concerns CRC patients/tissues; Akt—serine-threonine protein kinase; AMPK—AMP-activated protein kinase; AT—adipose tissue; C—control; CC—cancer cachexia; (F)FA—(free) fatty acid; FIS1—fission protein 1; FOXO—Forkhead-O; HMGB 1—high mobility group box 1; eIF4E—eukaryotic initiation factor-4E; IL-1, -6, -8—interleukin 1, 6, 8; IFN-γ—interferon gamma; MAPK—AMP-activated protein kinase; MAFbx—muscle atrophy F-box; MAPK—mitogen-activated protein kinase; MuRF1—muscle RING-finger 1; NF-κB—nuclear factor kappa B; PIF—proteolysis-inducing factor; pSTAT3—phosphorylated signal transducer and activator of transcription protein; ROS—reactive oxygen species; SDS-MYL1—SDS-soluble myosin light chain 1; SMI—skeletal-muscle index; SOCS3—suppressor of cytokine signaling 3; TG—triglycerides; TNF-α—tumor necrosis factor α; UPC1—uncoupling protein 1; UPS—ubiquitin-proteasome system; WAT—white adipose tissue; WSC—weight stable cancer.

**Table 2:** The tumor microenvironment (TME) cellular components demonstrated in in vitro and in vivo models of colorectal cancer (CRC) cachexia and the known signaling pathways involved in this process.

TME Cell Type	Secreted Mediators	Intercellular Cooperation	Signaling Pathways	Potential Role in Cachexia	Ref.
TAMs	**M1 cells**: ↑IL-6, IFN-γ, CXCL8/IL-8, CCL2/MCP1; **M2 cells**: ↑IL-6, CCL2; **Monocytes**: ↑IL-6, IL-12b, IFN-γ; IL-1; ↓IL-4, IL-10, TNF-α	Other immune cells (e.g., T cells); CRC cells (including colon adenocarcinoma C26 cells)	IL-1/IL-1R1; IL-6/STAT3; PGE2/EP;JAK2/STAT3/miR-506-3p/FOXQ1; IL-6R/STAT3/ miR-204-5p	(i) M1: pro-inflammatory in vitro and in vivo, promoting of type-1 T-cell response, with a tumor-suppressive role, and inhibiting of proliferation of tumor cells; (ii) ↑cellular interaction between IL-1R-expressing tumor cells and TAMs; (iii) M2: regulate of EMT, ↑migration and invasion of CRC cells, induce of chemoresistance of CRC cells; (iv) ↓TAMs number correlated with disturbed skeletal-muscle regenerative ability in CC through ↓CCL2-5, CXCL3 levels	[23,53,72,83,85,86,87,88,89,90]
TILs	**T cells**: ↑TNF-α, IL-6; **PBMCs**: ↑IL-6, IL-4, IL-10; ↓IL-12	Other immune cells (e.g., macrophages); CRC cells		(i) ↑tumor growth; (ii) ↑↑IL-6 correlated with ↓CD8+ and CD4+ lymphocytes, and ↑MDSCs and Treg/suppressor T cells in tumor; (iii) correlation between ↑↑IL-6 and ↑↑PD-L1; (iv) ↓intestinal-tumor immunosurveillance	[92,96,97,98]
TANs	↑TNF-α, IL-6, CXCL1, COX-2	Other immune cells (e.g., T cells); CRC cells	TGF-β; IFN-γ; PGE2/EP2	(i) EP2 stimulate TANs to ↑expression of cytokines/chemokines and other pro-inflammatory genes; (ii) role in angiogenesis, cell migration, invasion, and metastasis; (iii) ↑CD8^+^ T-cell activation, proliferation, and cytokine release; (iv) ↑CD45RO/CD62L T-cell number; (v) ↓TANs number correlated with disturbed muscle regenerative ability in CC through ↓CCL2-5, CXCL3 levels	[20,23,90,100,101,102,104]
MDSCs	↑TNF-α, IL-1, IL-6	Other immune cells (e.g., T cells); CRC cells		(i) ↓T cell functions after HIF-1α stimulation; (ii) promote CRC cell stemness and progression via exosomes enriched in S100A9; (iii) suppress cell-mediated immune response; (iv) circulating cells correlate with sIL-2R levels and nutritional parameters	[108,110,111,113]
CAAs	**pAT**: ↑TNF-α, STAT1, FADD; **sAT**: ↑TNF-α, IL-1β; CCL2/MCP-1 gene expression; ↑CCL3/MIP-1α, CCL4, IL-1β and TNF-β protein expression; ↑ATGL and HSL, ↓perilipin **AT and plasma**: ↑IL-8, TGF-β1, -β2, and β3; IFN-γ, EGF; GM-CSF, HIF-1α; ↑leptin, adiponectin, IGF1, TNF-α, IL-6, IL-8, IL-10, CCL2, VEGF	CRC cells; Cancer myofibroblasts; Immune cells (e.g., macrophages, lymphocytes)	TGF-β; PI3K/JAK/STAT3/ MAPK	(i) recruit of M1 and M2 macrophages; (ii) ↑lipolysis in sAT associated with macrophage infiltration (prevalent reparative inflammatory response), imbalance lipases expression; (iii) ↑of fibrosis of sAT, tumor remodeling, transdifferentiation of fibroblast to myofibroblasts surrounding adipocytes with ↑ECM (iv) imbalance inflammatory cytokine profile; (v) activate cell-cycle regulators; (vi) are reprogrammed into CAFs by cancer cells; (vii) ↑tumor cell growth and invasion	[18,29,30,117,118,120,121,122,123,125,126]
CAFs	↑CCL2, CCL8; FSTL1; IL-6; TNF-α, IL-5, IL-8; ADAMs; COX-2, Wnt genes; PGE2	CRC cells; CAAs; Immune cell (e.g., NK cells)	TGF-β/Smad; TNFR2/Akt (ERK); VEGF-A; PGE2/EP	(i) metabolically support tumor growth via the local stromal generation of mitochondrial fuels; (ii) role in tissue remodeling and fibrosis/desmoplakia; (iii) imbalance inflammatory cytokine profile; (iv) ↑migration/invasion of CRC cells; (v) role in angiogenesis (vi) ↑ECM synthesis; (vii)regulate expression of inflammation- and growth-related genes and suppress NK cells function	[23,29,30,127,135,138]
CRC cells	↑PIF; progranulin ↑IL-6, IL-6R, IL-1R1, IL-1β; ↑TNF-α; ↑↑Fn14 (receptor of TWEAK); ↑oncostatin M, LIF↑RAGE; ↑cachexokines (e.g., Ataxin-10)	Immune cells (e.g., monocytes/ macrophages, T cells, MDSCs); liver cells; CAAs; CAFs; ECs	JAK/STAT3; LIF/JAK2/STAT3	(i) ↑protein degradation; (ii) ↑IL-6 and IL-8 production by liver cells; (iii) ↑strong immunosuppression in TME and metastatic colonization of C26 cells via IL-6; (iv) role in muscle wasting, systemic inflammation, release of tumor-derived procachectic factors through RAGE, S100B and HMGB1; (v) ↑atrophy of cardiomyocytes and alter lipid metabolism; (vi) ↑myotubes atrophy; (vi) role in monocytes differentiation toward inflammatory phenotypes; (vii) role in activation of fibroblast to myofibroblasts; (viii) ↑IL-6 production from fibroblasts resulting in ↑integrin β6; (ix) ↑glycolysis in CAFs and ECs, cause T-cell immunosuppression	[53,89,97,118,132,134,136,139,140,142,143,144,146,147,148,149,151]

**Legend**: ↑, ↓—increase/decrease expression/level; ↑↑—overexpression; (s/p)AT—(subcutaneous/peritumoral) adipose tissue; ATGL—adipose triglyceride lipase; CAAs—cancer-associated adipocytes; CAFs—cancer-associated fibroblasts; CC—cancer cachexia; CCL2—the chemokine (C-C motif) ligand 2; MCP-1—monocyte chemo-attractant protein 1; CCL3—the chemokine ligand 3/macrophage inflammatory protein-1 α (MIP-1α); COX-2—cyclo-oxygenase-2; CXCL8—the chemokine (C-C motif) ligand 8/IL-8; ECs—endothelial cells; ECM—extracellular matrix; EMT—epithelial-mesenchymal transition; FADD—FAS-associated death domain; FOXQ1—Forkhead box Q1 protein; FSTL1—follistatin-related protein 1; GM-CSF—granulocyte-macrophage colony-stimulating factor; HMGB1—high mobility group box 1; HSL—hormone-sensitive lipase; IFN-γ—interferon gamma; (IL-) 6R—(interleukin) 6 receptor; JAK2—Janus kinase 2; LIF—leukemia inhibitory factor; M1/M2—phenotypes of macrophages; MAPK—mitogen-activated protein kinase; MDSCs—myeloid-derived suppressor cells; miR—microRNA; PBMCs—peripheral blood mononuclear cells; PD-L1—programmed death-ligand 1; PGE2/EP2—prostaglandin E2 receptor 2 subtype; PI3K—phosphatidylinositol 3 kinase; pM/pT—pathological metastasis/tumor size; RAGE—receptor for advanced glycation end-products; STAT1, -3—signal transducer and activator of transcription protein 1, 3; TAMs—tumor-associated macrophages; TANs—tumor-associated neutrophils; TGF-β—tumor growth factor beta; TILs—tumor-infiltrating lymphocytes; TME—tumor microenvironment; TNF-α—tumor necrosis factor α; TWEAK—TNF-like weak inducer of apoptosis; VEGF—vascular endothelial growth factor.

**Table 3:** Therapeutical options in the management of cancer cachexia in solid cancers (including CRC).

Name of Targeted Agents	Agent/Phase	Population/Animal Model of the Study	Main Effects in Cachexia	Ref.
Thalidomide	a derivative of glutamic acid; phase II	Different advanced human cancer types; (i) megestrol acetate (MA) plus thalidomide; (ii) megestrol	MA + thalidomide improved weight, fatigue, quality of life, grip strength, GPS, ECOG performance status; ↓IL-6, and TNF-α in combination arm	[176]
MABp1	first-in-class true human IgG1k MAb against IL-1α; phase III	humans	(i) antitumor; (ii) more patients in MABp1 group achieved the composite primary outcome; (iii) MABp1 group had lower IL-6, less thrombocytosis, and longer survival	[174]
Anamorelin (ONO-7643; ANAM)	ghrelin receptor agonist; approved for use in Japan (2020)	humans	(i) improves anorexia and patients’ nutritional status; (ii) ↑serum IGF1	[181,182]
**Promising trials on animal models**				
GH, insulin, indomethacin		C26 model, mouse model	alleviated tumor-free body-weight reduction and CC-induced changes in nutritional markers and cytokines, and prolonged survival time	[172]
Anti-Fn14	antibody against Fn14	C26 cells, mice, humans	(i) retain body mass; (ii) retain muscle mass, (iii) retain AT	[146]
Anti-RAGE	antibody against RAGE	mouse model	lack of RAGE results in reduced serum levels of cachexia-inducing factors, delayed loss of muscle mass and strength, reduced tumor progression, and increased survival	[147]
CDD866	a neutralizing antibody against ActRII	CT-26 mouse colon cancer-induced cachexia model	administration of CDD866 alone or in combination with cisplatin protected from skeletal-muscle weight loss compared to animals receiving only cisplatin	[183]
Resveratrol	natural phytoalexin	C26 cells, mouse model	(i) inhibits NF-κB in cancer cells; (ii) inhibits of NF-κB activity in skeletal and heart muscle; (iii) inhibits skeletal and cardiac muscle atrophy induced by C26 tumors	[185]
*Citrus unshiu* Peel Extract (CUPE)	traditional herbal drug	C26 tumor-bearing BALB/c male mice	(i) ↓weight loss, tumor volume, and serum MDA levels; (ii) the combination therapy (CUPE + Dox) leads to ↓↓serum levels of IL-6, TNF-α, IL-1β and tumor volume vs. untreated tumor-bearing mice and Dox groups	[184]

**Abbreviations:** ↑, ↓—increase (up-regulation)/decrease expression/level; ActRII—activin receptor type II; AT—adipose tissue; CC—cancer cachexia; Dox—Doxorubicin; ECOG—Eastern Cooperative Oncology Group GH- growth hormone; GPS—Glasgow prognostic score; IGF1—insulin-like growth factor 1; MDA—malondialdehyde-thiobarbituric acid; NF-κB—nuclear factor kappa B; RAGE—receptor for advanced glycation end-products; TNF-α—tumor necrosis factor α; IgG1k—monoclonal antibody (MAb); IL-1, -6, etc.—interleukin 1, -6, etc.

## Abbreviations

AAs	Amino Acids
ACC	Acetyl-CoA Carboxylase
ADAMs	A Disintegrin and Metalloprotease
Akt	Serine/threonine protein kinase
AMPK	AMP-activated Protein Kinase
APC	Adenomatous Polyposis Coli
AT/WAT	Adipose Tissue/White AT
ATGL	Adipose Triglyceride Lipase
CAAs	Cancer-associated Adipocytes
CAFs	Cancer-associated Fibroblasts
C26	Colon Adenocarcinoma 26 Cells
CC	Cancer Cachexia
CCL2/MCP-1	Chemokine (C-C Motif) Ligand 2/Monocyte Chemoattractant Protein 1
CCL3/MIP-1α	Chemokine (C-C Motif) Ligand 3/Macrophage Inflammatory Protein-1 α
CD3, -4, -8	Cluster of Differentiation 3, -4, -8
CIFs	Cachexia-inducing Factors
COX-2	Cyclooxygenase-2
CPT-1	Carnitine Palmitoyltransferase 1
CRC	Colorectal Cancer
CRP	C-reactive Protein
CSS	Cancer-specific Survival
CXCL8/IL-8	Chemokine (C-X-C Motif) Ligand 8/Interleukin 8
DCs	Dendritic Cells
DFS	Disease Free Survival
ECs	Endothelial Cells
ECM	Extracellular Matrix
EGF	Epidermal Growth Factor
eIF2	Eukaryotic Initiation Factor 2
EMT	Epithelial-Mesenchymal Transition
ERK	Extracellular-regulated Kinase
FADD	FAS-associated Death Domain
FFA	Free Fatty Acid
Fn14	Receptor of the TWEAK
FOXQ1	Forkhead Box Q1 Protein
FSP1	Fibroblast-specific Protein 1
FSTL1	Follistatin-related Protein 1
G(M)-CSF	Granulocyte(Macrophage)-Colony-stimulating Factor
GPIHBP1	Glycosylphosphatidylinositol-anchored High-density Lipoprotein Binding Protein 1
HIF-1α	Hypoxia-inducible Factor 1 alpha
HMGB1	High Mobility Group Box 1
HSL	Hormone-sensitive Lipase
IFN-α, β, γ	Interferon alpha, beta, gamma
IGF1	Insulin-like Growth Factor 1
s(IL-)2R, IL-1R1	Soluble (Interleukin) 2 Receptor; IL-1Receptor type I
IP-10	Interferon gamma-induced protein 10/CXCL10
JAK2	Janus Kinase 2
LIF	Leukemia Inhibitory Factor
LMR	Lymphocyte:Monocyte Ratio
mABs	Monoclonal Antibody
MAFbx	Muscle Atrophy F-box
MAPK	Mitogen-activated Protein Kinase
MDSCs	Myeloid-derived Suppressor Cells
MEK1/2	Mitogen-activated Protein Kinase (MAP2K, MEK, MAPKK)
mGPS	Modified Glasgow Prognostic Score
MLNs	Mesenteric Lymph Nodes
MSI-H	High Microsatellite Instability
MSS	Microsatellite Stability
mTORC1	Mammalian Target of Rapamycin Complex 1
MuRF1	Muscle RING-finger 1
MyoD	Myoblast Determination Protein 1
NF-κB	Nuclear Factor kappa B
NK	Natural Killer Cells
NLR	Neutrophil:Lymphocyte Ratio
NOS2	Nitric Oxidase Synthase 2
OS	Overall Survival
PBMCs	Peripheral Blood Mononuclear Cells
PD-L1	Programmed Death-Ligand 1
PGE2/EP2	Prostaglandin E2 Receptor 2 Subtype
PIF	Proteolysis-inducing Factor
PI3K	Phosphatidylinositol 3 Kinase
PP	Peyer’s Patches
RAGE	Receptor for Advanced Glycation End-products
RANTES	Regulated on Activation, Normal T-cell Expressed and Secreted
RFS	Recurrence-free Survival
ROS	Reactive Oxygen Species
SDS-MYL1	Sodium Dodecyl Sulfate Soluble Myosin Light Chain 1
SII	Systemic Inflammatory Index
SIR	Systemic Inflammatory Response
SMI	Skeletal-Muscle Index
SOCS3	Suppressor of Cytokine Signaling 3
SSAT	Spermidine/Spermine N-1 Acetyl Transferase
STAT1, -3	Signal Transducer and Activator of Transcription Protein 1, 3
TAMs	Tumor-associated Macrophages
TANs	Tumor-associated Neutrophils
TGF-β	Tumor Growth Factor beta
TIICs	Tumor-infiltrating Immune Cells
TILs	Tumor-infiltrating Lymphocytes
TME	Tumor Microenvironment
TNF-α, TNFR2	Tumor Necrosis Factor α/TNF Receptor 2
Treg	Regulatory T Cells
TWEAK	TNF-like Weak Inducer of Apoptosis
UPC1	Uncoupling Protein 1
UPS	Ubiquitin-proteasome System
VEGF-A	Vascular Endothelial Growth Factor A
WBC	White Blood Cells
Wnt	Wnt (gene wingless + integrated or int-1) Family Member
ZAG	Zinc-α2-Glycoprotein
